# Impact of heart rate variability-based exercise prescription: self-guided by technology and trainer-guided exercise in sedentary adults

**DOI:** 10.3389/fspor.2025.1578478

**Published:** 2025-05-22

**Authors:** Antonio Casanova-Lizón, Agustín Manresa-Rocamora, José Manuel Sarabia, Diego Pastor, Alejandro Javaloyes, Iván Peña-González, Manuel Moya-Ramón

**Affiliations:** ^1^ Department of Sport Sciences, Sport Research Centre, Miguel Hernandez University, Elche, Spain; ^2^ Alicante Institute for Health and Biomedical Research (ISABIAL), Alicante, Spain

**Keywords:** app, healthy sedentary people, heart rate variability, physical exercise, physical fitness, wearable technologies

## Abstract

**Introduction:**

Exercising at home is an accessible alternative to the gym, although it presents challenges such as low adherence, poor quality and difficulties in reaching set goals. Wearable technologies and the use of heart rate variability (HRV) make it possible to personalise workouts, optimise fitness and improve adherence. However, specific exercise recommendations based on these metrics are still lacking. This study evaluated the impact of HRV-based training using the Selftraining UMH app in an autonomous format versus a Personal Trainer-led approach.

**Methods:**

Seventy sedentary adults were divided into three groups: Autonomous (*n* = 18), Personal Trainer (*n* = 23), and Control (*n* = 29). After a two-week baseline HRV assessment, participants underwent an 11-week intervention, with pre- and post-tests on peak oxygen uptake, aerobic power, total test time, strength, and HRV.

**Results:**

Both intervention groups completed similar session numbers (23.3 vs. 24.5) and high-intensity workouts (13.7 vs. 14.6). Both groups improved significantly (*p* < 0.05) across all fitness metrics, except aerobic power in the Autonomous group. Effect sizes ranged from small to large (0.21–1.12 Autonomous; 0.23–1.63 Personal Trainer). Strength improvements were greater in the Personal Trainer group, and both outperformed the Control group (*p* < 0.05) on all variables except aerobic power in the Autonomous group.

**Conclusions:**

The findings demonstrate that HRV-based training effectively enhances fitness in sedentary adults, with both delivery methods showing similar adherence and benefits. The Selftraining UMH app offers an accessible alternative for autonomous exercise, particularly in settings without professional supervision, promoting improved population health outcomes.

## 1 Introduction

Physical exercise is a cornerstone of overall health, offering benefits beyond muscle conditioning and disease prevention. Scientific evidence links physical inactivity to non-communicable diseases, such as stroke, hypertension, type 2 diabetes, obesity, coronary heart disease, certain cancers, dementia, mental health problems, and increased mortality rates, particularly from cardiovascular disease (1). Conversely, regular exercise not only helps combat these conditions but also improves cognitive function (2) and promotes well-being (3). Despite its benefits, modern society has experienced increasing levels of sedentary behaviour (4). In 2016, physical inactivity was more than twice as prevalent in high-income countries (36.8%) compared to low-income countries (16.2%), with an upward trend observed in wealthier nations between 2001 and 2016 (5).

Traditionally, exercise has been associated with gyms and sports facilities, but many individuals face barriers such as time constraints, financial limitations and accessibility issues (6). Recent research highlights the benefits of home exercise as a more convenient and accessible alternative (7), promoting well-being (8), cardiovascular (9) and mental health (10). However, lack of supervision in home exercise programmes can lead to challenges such as inconsistent adherence, poor exercise quality, and difficulty achieving fitness goals (11). Adherence, defined as following professional recommendations (12), is influenced by personal and environmental factors (13). In addition, there is no universal standard for assessing exercise adherence, complicating efforts to identify predictors of success (13).

The rise of digital technologies, especially wearable technologies (WTs), offers new solutions for objective measurement and guiding exercise (13). WTs, such as smartwatches and devices embedded in smartphones, enable real-time data collection and feedback (14). These devices have been shown to improve adherence by providing users with objective measurements and education (15). Increasing emphasis is being placed on a personalised approach to exercise, recognising the variability in individuals' physiological responses to training (16). Day-to-day training models dynamically adjust workouts based on personal data, optimising performance and reducing the risk of injury (17).

Heart rate variability (HRV) has emerged as a key tool for personalising training. HRV measures variations between consecutive heartbeats and reflects the balance between the sympathetic and parasympathetic branches of the autonomic nervous system (ANS) (18). HRV is sensitive to exercise response and fatigue levels (19, 20), making it a valuable indicator for adjusting training intensity. For example, normal values of HRV suggest readiness for intense exercise, while altered values indicate the need for lighter activity or rest (11). Validated applications such as HRV4Training (21) and Welltory (22), along with devices such as Elite HRV (23), offer accessible ways to measure HRV. However, these tools typically provide HRV values without specific exercise recommendations based on those metrics.

Due to the current challenges in accessing guided training, the efficacy of HRV as an exercise control tool, and the role of personal training in measuring this variable, the Miguel Hernández University of Elche has developed a mobile application called Selftraining UMH. This application employs a day-to-day guided model based on HRV. Therefore, the objective of this study was to analyse adherence and the effects of self-directed training prescribed using the Selftraining UMH app, as well as training led by a personal trainer.

## 2 Materials and methods

### 2.1 Participants

Participants were male and female adults, either sedentary or with at least three months of physical inactivity, willing to start a physical exercise programme in an autonomous or a personal trainer-led mode. Exclusion criteria included pre-existing medical conditions, age over 60, or being physically active (engaging in >150 min of moderate-intensity or >75 min of vigorous-intensity activity per week) (24). The information required to verify the inclusion and exclusion criteria was self-reported through an initial interview and the Physical Activity Readiness Questionnaire (PAR-Q) (25).

In the initial phase of the study, recruitment of participants was carried out through a multi-channel strategy. Various dissemination platforms were used, including project- and institution-specific social networks, information posters and leaflets distributed, as well as awareness-raising campaigns through local radio stations. In parallel, participation was promoted through informative talks given in university classrooms and educational centres.

Participants provided written informed consent, and ethical approval was granted by the university's Ethics Committee (CID.DPC.01.19), adhering to Good Clinical Practice and the Declaration of Helsinki.

### 2.2 Experimental design

**Figure 1:**
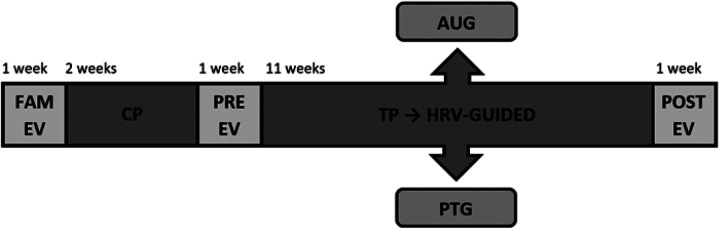
Experimental design. AUG, autonomous group; FAM-EV, familiarization evaluation; CP, control period; PRE-EV, evaluation conducted prior to the training period; PTG, personal training group; TP, training period; HRV, heart rate variability guided; POST-EV, evaluation after the training period.

The study spanned 16 weeks, divided into two periods: a 2-week control period (CP) for stabilisation and baseline HRV measurements, followed by an 11-week training period (TP). Assessments were conducted before (PRE-EV) and after the training period (POST-EV), with a familiarization week (FAM-EV) to minimize the learning effect. Participants who were not interested in the training programme were not randomised and, therefore, assigned to the control group (CG). The remaining participants were randomised into two groups: an autonomous HRV-guided group using the Selftraining UMH app (AUG) or a personal trainer-led HRV-guided group (PTG). HRV was used to prescribe daily training (Figure 1).

### 2.3 Evaluation

#### 2.3.1 Physical fitness variables

The evaluations were conducted in the Training Analysis and Optimization Laboratory of the Sport Research Centre of the Miguel Hernández University of Elche. Participants refrained from drinking or speaking during the test and avoided intense exercise 24 h prior. Tests included:

Aerobic Capacity: peak oxygen uptake (VO_2_ peak) was measured via an incremental treadmill test using the Bruce protocol (26), with gas exchange data collected using the Metalyzer 3B (Cortex GmbH, Germany).

Upper Body Strength: A one-minute Bench Press Test was performed using a Smith Machine with a standardised load (35 kg for men, 15 kg for women) (27). Proper execution required full elbow extension during the upward phase and allowing the bar to touch the chest during the downward phase.

Lower Body Strength: A squat test assessed maximum repetitions in one minute, ensuring proper form and a consistent depth (27). A brief warm-up was conducting before starting the test. During this phase, participants were instructed on proper squatting technique. A box was positioned to provide a consistent depth, ensuring 90 degrees of knee flexion at the bottom of each repetition (28). Participants received feedback on their technique throughout the test. Repetitions that performed incorrectly were excluded from the total count.

#### 2.3.2 Assessment of heart rate and HRV

Heart rate (HR) and HRV were recorded using a Polar H10 chest strap and the Elite HRV app (29). During the subsequent 5 min, HR and HRV data were recorded while participants remained seated in a resting state. The final 2 min of the recording were earmarked for subsequent analysis (30) using Kubios HRV Premium software (v3.5.0) (31). The root mean square of successive differences (rMSSD) parameter was chosen due to its reliability (32), and ectopic beats were corrected using automated artefact correction in the Kubios software (33).

#### 2.3.3 Adherence to exercise training

Adherence for the PTG was tracked via attendance records (34), and for the AUG, through app usage logs. HR monitors verified target HR zones. Across the 11 weeks, participants were scheduled for 33 sessions, with adherence monitored to ensure compliance.

Training zones were determined based on the percentage of maximum heart rate (HR max), following the classifications established by the American College of Sports Medicine (35). In this study, high-intensity exercise was defined as activity at ≥80% of HR max, moderate-intensity exercise ranged from 60% to 79% of HR max, and low-intensity exercise was classified as ≤59% of HR max.

#### 2.3.4 SF-36 health survey

This self-administered questionnaire assessed eight health domains, scored on a 0–100 scale, with higher scores indicating better health-related quality of life (36, 37). The Spanish adaptation by Alonso et al. (38) was used in this study.

#### 2.3.5 Psychological vitality and affective state

Psychological vitality was measured using the Subjective Vitality Questionnaire (39), with responses recorded on a 0–7 Likert scale. The Spanish adaptation by Molina-García et al. (40).

The Positive and Negative Affect Schedule (41, 42) measured emotional states before and after exercise sessions, using a nine-item questionnaire with a 1–7 Likert scale. The Spanish adaptation by López-Gómez et al. (43).

### 2.4 Physical exercise programme

The programme lasted 11 weeks, with a frequency of three sessions per week, each lasting 45–60 min, with 48–72 h of rest between sessions. Both the mobile app and the supervised programme included four levels of intensity, each offering four different session options. The first level included low-intensity sessions, and the remaining levels (level 2, level 3 and level 4) included high-intensity sessions (11). The training programme was gamified through the app to enhance motivation and participation. Participants earned stars for completing sessions within a level. Once a predetermined number of stars were accumulated, they progressed to the next level of intensity. Designed to span three months, the app provides a comprehensive, adaptable and user-friendly framework for people starting an exercise routine, ensuring sustained engagement and progressive development. This design ensured a gradual increase in intensity and workload, while maintaining a balance between challenge and adaptability to suit participants' abilities. Sessions included warm-up, strength and endurance exercises, as well as a cool-down phase.

In level 1, the main objective was to familiarise participants with the physical exercise programme and ensure proper technique. Strength exercises included squats, shoulder presses, lunges, push-ups and rowing. In addition, core-focused exercises such as front planks, side planks, dynamic and static back bridges, dead bugs, and bird dogs were introduced. Each exercise consisted of 15 repetitions, followed by one minute of rest. During rest, participants alternated between 20 s of core exercises (e.g., active planks) and 40 s of passive rest. Participants completed four sets of each exercise before moving on to the next, performing 4–5 exercises per session. A two-minute rest was provided between exercises (Supplementary Figure S1). In level 2, the intensity was increased by incorporating additional aerobic exercises alongside the strength exercises from level 1. Aerobic activities included mountain climbers, skipping, jumping jacks, burpees, side steps, and boxing. Each exercise was performed for 30 s, followed by a 30 s rest. Sessions consisted of 10–12 exercises per set, and participants completed two sets in total (Supplementary Figure S2). In level 3, the integration of aerobic and strength exercises was maintained while the training volume was increased. Participants performed three sets instead of two. The number of exercises per session started with 8 during the first weeks and increased to 10 in the following weeks. The duration of each exercise (30 s) and the rest period (30 s) remained unchanged. A two-minute rest interval was introduced between sets (Supplementary Figure S3). In level 4, the structure of level 3 was maintained, but the work-rest ratio was modified to increase the intensity. Each exercise was performed for 40 s, while the rest period was reduced to 20 s. The two-minute rest interval between sets remained unchanged, and the number of exercises and sets was consistent with that of level 3 (Supplementary Figure S4).

The programme started with low-intensity sessions in weeks 1 and 2 and progressed to two high-intensity and one low-intensity session per week. All sessions were designed so that they could be performed at home or outdoors, using minimal or low-cost equipment to ensure accessibility. The number of intervals increased gradually throughout the programme, following a linear periodisation approach. This strategy facilitated a progressive increase in high-intensity exercise, starting with a low volume to promote safe physiological adaptation. The periodisation plan and the evolution of training variables can be found in Supplementary Table S1. The exercise programme was developed based on fundamental training principles, including overload, progression, individualisation, periodisation and specificity (44).

### 2.5 HRV-guided training prescription

Participants recorded HRV daily using the Welltory smartphone app (22) and photoplethysmography (PPG) (45). Measurements, lasting two minutes, were taken in a supine position upon waking, with a consistent breathing pattern. HRV values were transformed into their natural logarithms (Ln-rMSSD) for parametric analysis (46). Training prescriptions were based on a seven-day rolling average (Ln-rMSSD_7day−roll−avg_) relative to the smallest worthwhile change (SWC), defined as the mean ± 0.5 × standard deviation (SD) (47).

**Figure 2:**
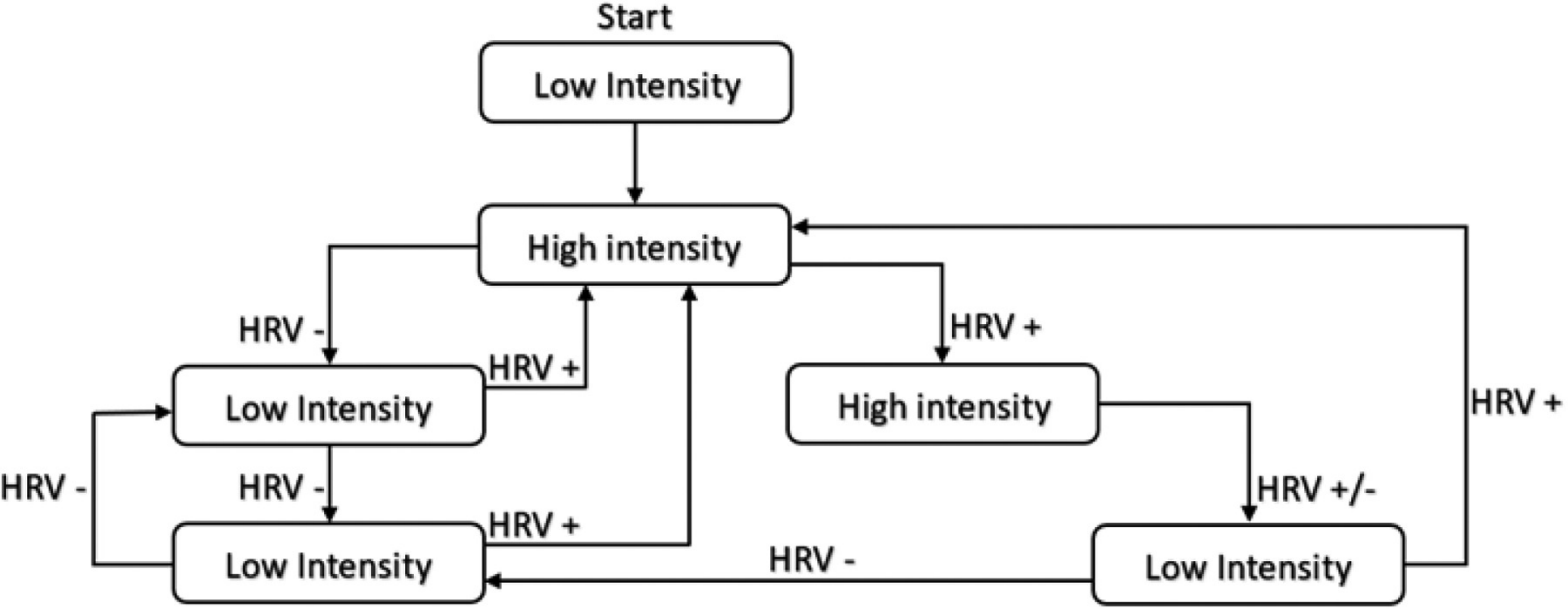
HRV-guided training decision algorithm.

Training intensity was guided by the Ln-rMSSD_7day−roll−avg_ relative to the SWC. High-intensity sessions were prescribed when HRV was above or within the SWC, while low-intensity sessions were recommended if HRV was below the lower limit of the SWC (48, 49). A variation of the decision-making algorithm developed by Javaloyes et al. (48) was followed, which is a modification of the algorithm proposed by Kiviniemi et al. (49) (Figure 2). Supplementary Table S2, details how progress was made at different intensity levels throughout the training programme for both experimental groups, where HRV was within normal HRV values as determined by the SWC. Supplementary Table S3 presents the evolution of the training programme over several weeks, establishing a differentiation in the intensity levels and their distribution according to the PNS response measured through the HRV. The progression of intensity is implemented in a gradual and controlled manner, ensuring adequate physiological adaptation before progressing to higher levels. The adopted model contemplates three high-intensity levels, which require the completion of six sessions within the same level, with HRV values above or within the SWC before allowing progression to the next level.

#### 2.5.1 Autonomous group guided by the Selftraining UMH app

The AUG followed the same programme remotely, using the Selftraining UMH app. Instructional videos provided guidance on app use and HRV measurement techniques (links can be found in the Supplementary Material). Weekly follow-ups via email or phone addressed technical issues and provided feedback on training adherence and execution.

#### 2.5.2 Group guided by a personal trainer

Participants in the PTG trained in person under supervision of sports science professionals at the Sports Research Centre of Miguel Hernández University of Elche.

#### 2.5.3 Control group

Prior to the study, and during short personal interviews, participants assigned to the CG were asked not to initiate any exercise programme throughout the study duration. Therefore, these participants maintained their previous physical activity levels. Afterwards, this information was confirmed during the final assessment. All the participants confirmed that they remained inactive during the full study period.

### 2.6 Structure and functionalities Selftraining UMH application

The Selftraining UMH mobile application is designed to prescribe physical training through the daily monitoring of HRV. The application is organised into three main sections. From the home screen, the user can access tools for monitoring their physiological state, including.

**Figure 3:**
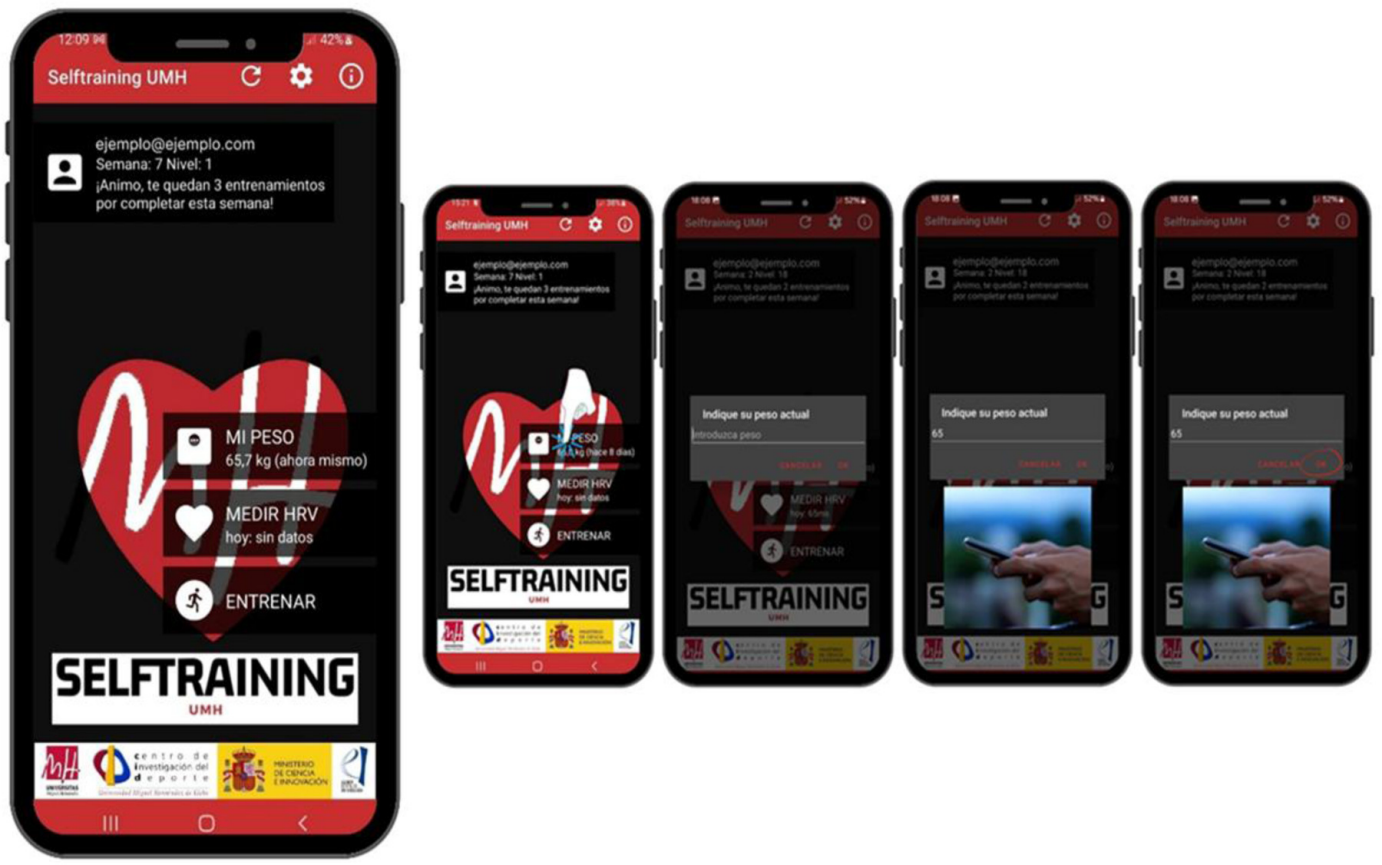
Manual recording of body weight.

The application enables the manual recording of body weight, allowing for continuous monitoring of physical condition over time. This feature facilitates the assessment of weight fluctuations and their potential impact on overall health and fitness (Figure 3).

**Figure 4:**
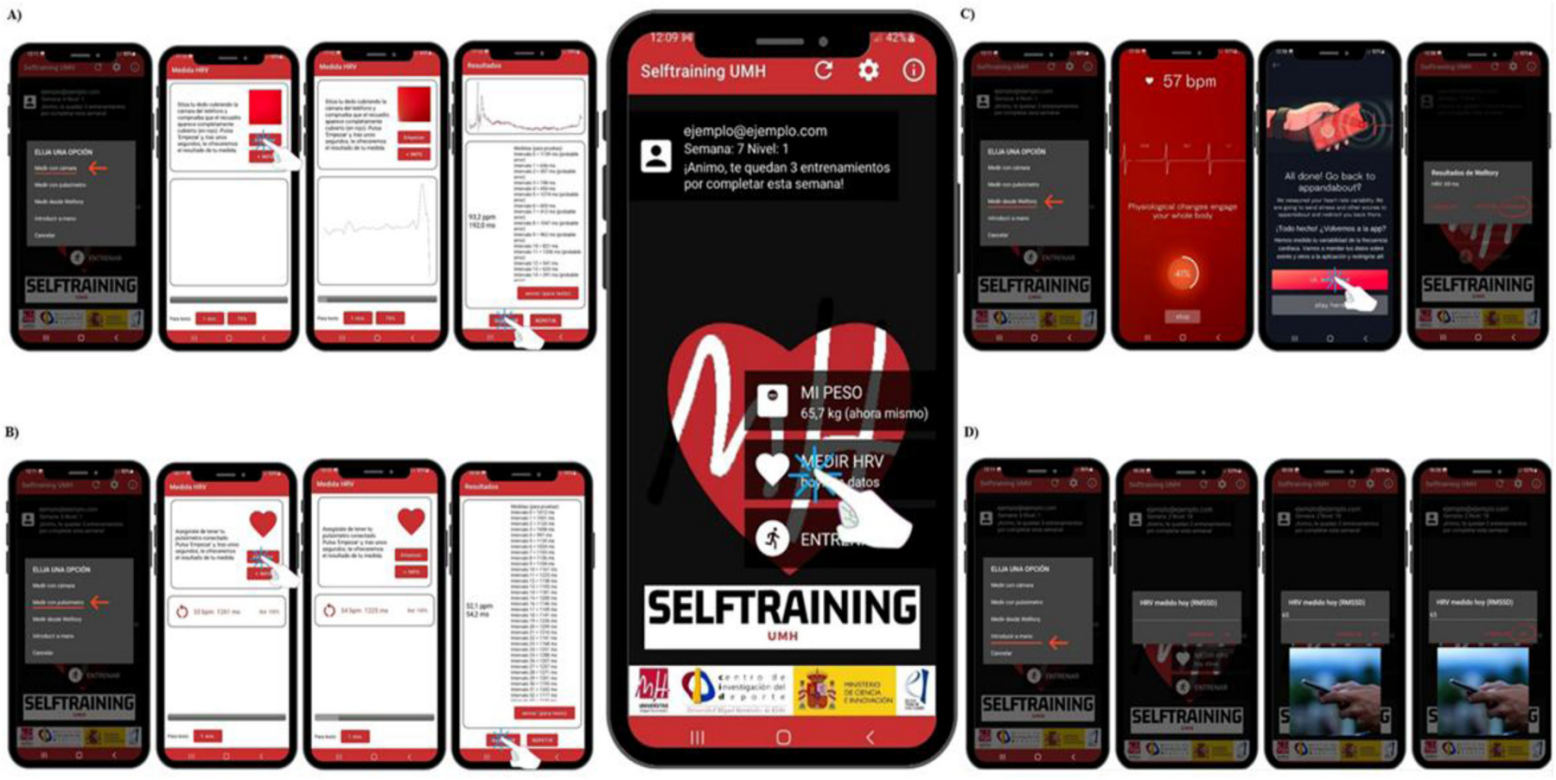
Measurement HRV various methods.

HRV is recorded each morning, providing physiological insights and generating personalised recommendations based on the obtained values. The measurement process can be conducted through various methods. When using optical measurement via a smartphone camera, the user follows on-screen instructions, positioning a finger over the camera and flash. The measurement is initiated by pressing the “START” button, leading to a one-minute recording. An additional informational section (“+INFO”) provides guidance on the correct procedure, the optimal conditions for measurement, and the recommended user's state. For HRV measurement using an external HR monitor, a compatible device must be connected. If used for the first time or not automatically detected, the user must manually enter the monitor's identification number. The measurement begins upon selecting the “START” button and lasts for one minute, with an informational section (“+INFO”) available for further instructions on the correct measurement protocol. Alternatively, HRV can be recorded via integration with the Welltory application, which requires permission to access relevant data. Upon launching the Welltory app, the user has six seconds to position the index finger over the camera and flash. The measurement process takes between one and two minutes, depending on movement and sensor coverage. Once completed, selecting the red button returns the user to the Selftraining UMH application, where the recorded HRV data can be saved. Additionally, the system allows for manual data entry, enabling the incorporation of HRV values obtained from other devices or applications, ensuring broader compatibility with external monitoring systems (Figure 4).

**Figure 5:**
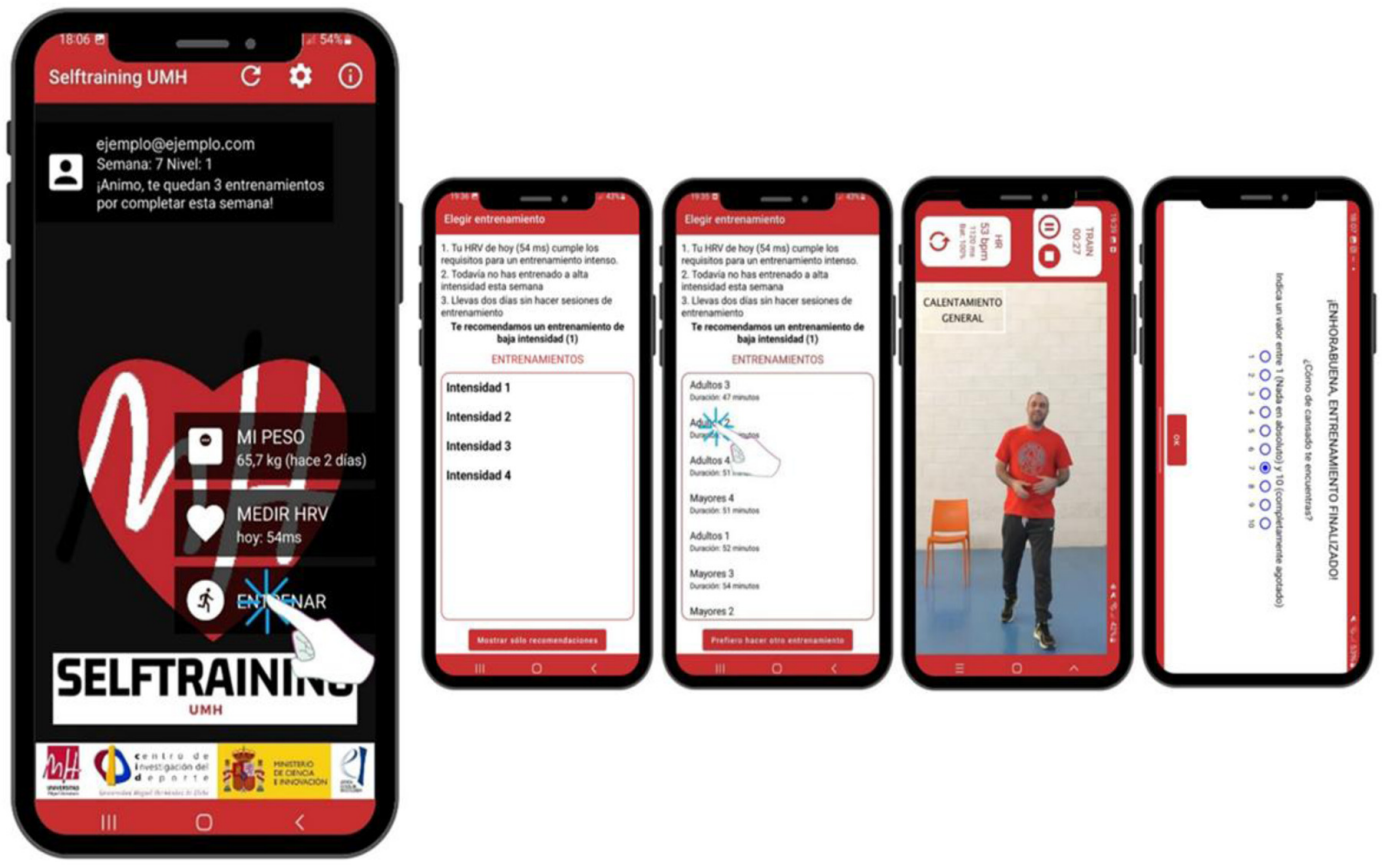
Training within the application.

The application includes a structured autonomous training programme designed based on physiological data. This programme spans three months and consists of three weekly training sessions, each lasting one hour. The intensity of each session is determined through a proprietary algorithm that analyses previously recorded HRV values, allowing for a personalised approach to training. A comprehensive library of pre-recorded video workouts is accessible within the application, offering users a variety of training options. Prior to each session, the user is provided with information regarding their physiological state and recommended intensity levels. While the application generates optimised workout suggestions, flexibility is offered through the option to select sessions of varying intensity. During training, real-time performance tracking is available, with functionalities that allow the user to pause, stop, or restart the session. If a compatible HR monitor is connected, HR data can be recorded, further enhancing the precision of training adaptation. Following each session, a post-exercise self-assessment tool enables the user to evaluate their subjective fatigue levels using a numerical scale from 1 to 10, contributing to a more comprehensive understanding of physiological responses to exercise (Figure 5).

### 2.7 Training load quantification

Training load was assessed using the Edwards Training Impulse (eTRIMP) method (50), modified to include Zone 0 (<50% HR maximum) combined with Zone 1. The internal load was measured via the Session Rating of Perceived Exertion (sRPE) scale (51).

### 2.8 Statistical analysis

A repeated measures analysis of variance (ANOVA) was performed to analyse changes in each of the groups and an independent measures ANOVA was performed to compare exercise adherence between the two experimental groups. Finally, an analysis of covariance (ANCOVA) was used to analyse differences between changes after the intervention taking into account pre-intervention values, to reduce error variance and improve statistical power (52). Effect sizes (ES) Cohen's *d* were calculated with 95% confidence intervals and significance was set at *p* < 0.05, with the following interpretations: trivial (≤0.19), small (0.20–0.49), moderate (0.50–0.79), and large (≥0.80) (53). The association between variables was assessed with Pearson's correlation coefficient (*r*) and interpreted as trivial (≤0.09), small (0.10–0.29), moderate (0.30–0.49), high (0.50–0.69), very high (0.70–0.89) and almost perfect (≥0.90) (54). Analyses were performed using Excel and JASP software (55).

## 3 Results

### 3.1 Participants

**Figure 6:**
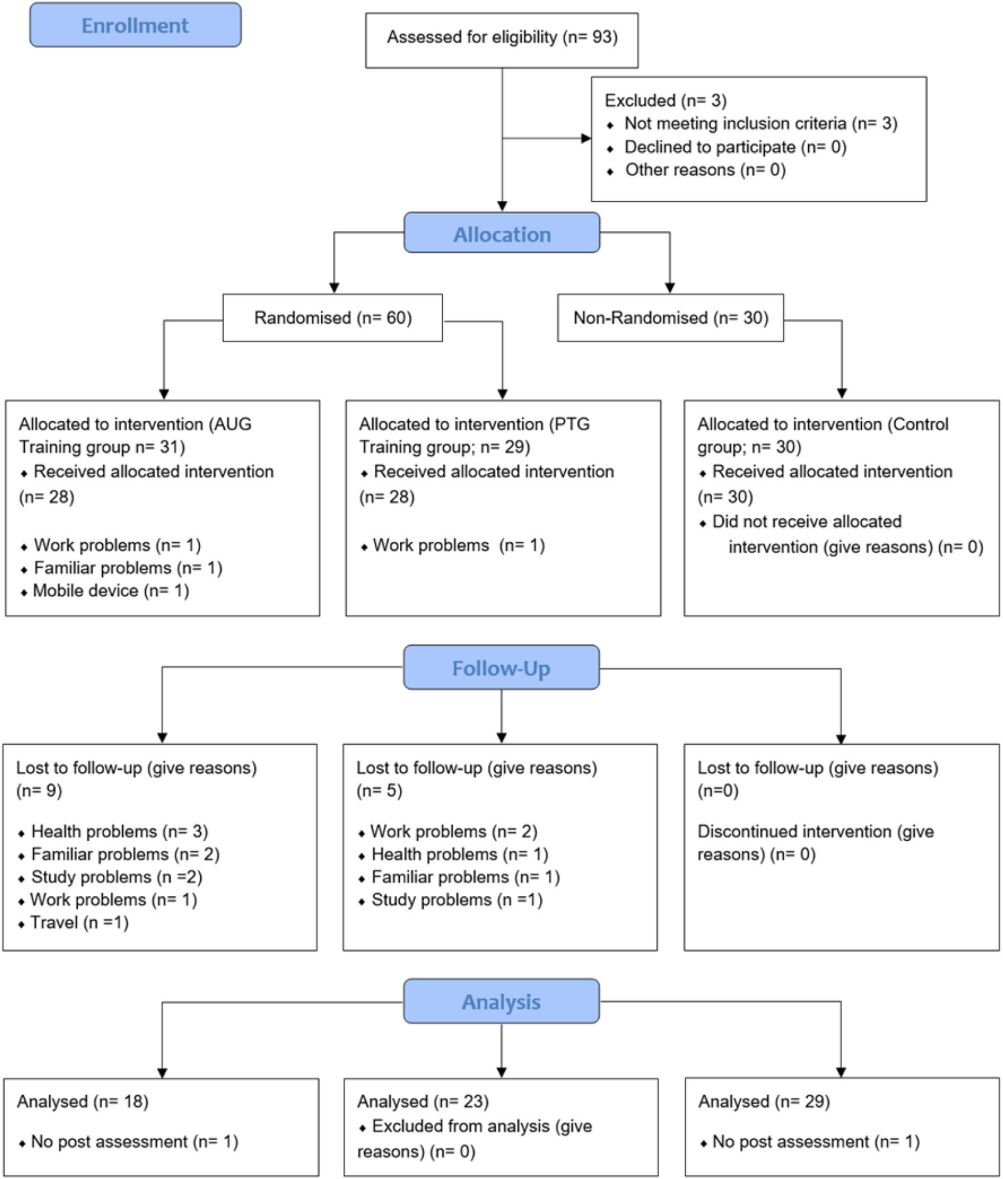
CONSORT 2010 flow diagram subjects. AUG, autonomous group; PTG, personal training group.

Ninety-three participants were recruited for the study. Three were excluded after the initial interview for not meeting the inclusion criteria (one for cardiovascular disease and two for being physically active), leaving 90 eligible subjects. These were randomly assigned to two experimental groups: AUG (*n* = 31) and PTG (*n* = 29), as the participants included in the CG (*n* = 30) were allocated by convenience. Four subjects did not start the assigned intervention: three from the AUG (9.7%, for work, family or technical reasons) and one from the PTG (3.4%, for work reasons). During the study period, 14 participants dropped out: nine from AUG (29%) and five from PTG (17.2%), mainly due to health problems, family, work or academic commitments. In addition, two participants did not attend the final evaluations: one from AUG (3.2%) and one from the CG (3.3%). In total, 70 subjects completed the 11-week programme: 18 in the AUG, 23 in the PTG and 29 in the CG. A detailed flow of participants' progress is presented in Figure 6 (56), and baseline descriptive characteristics are provided in Table 1.

**Table 1:** Descriptive statistics of adult participants (mean ± SD).

Characteristics	AUG (*n* = 18)	PTG (*n* = 23)	CG (*n* = 29)
Gender (male/female)	8/10	6/17	9/20
Age (years)	40.50 ± 9.30	37.41 ± 12.53	36.89 ± 10.97
Height (m)	1.67 ± 0.09	1.64 ± 0.08	1.67 ± 0.11
Weight (kg)	71.06 ± 11.34	67.33 ± 11.85	73.08 ± 18.35
BMI (kg·m^−2^)	25.65 ± 3.87	25.15 ± 4.41	26.23 ± 5.24
SBP (mmHg)	124.05 ± 13.52	116.44 ± 8.75	117.97 ± 13.07
DBP (mmHg)	78.68 ± 7.36	76.35 ± 7.36	74.97 ± 8.67
HR_rest_ (bpm)	71.32 ± 10.71	72.22 ± 14.37	70.53 ± 10.11
HR_max_ (bpm)	176.26 ± 18.45	180.61 ± 10.09	179.57 ± 13.47
Ln-rMSSD (ms)	2.89 ± 0.68	3.09 ± 0.83	2.88 ± 0.59

AUG, autonomous group; bpm, beats per minute; BMI, body mass index; CG, control group; DBP, diastolic blood pressure; HR_max_, maximum heart rate; HR_rest_, resting heart rate; Ln-rMSSD, natural logarithm natural logarithm root mean square of successive differences; *n*, sample number; PTG, personal trainer group; SMP, systolic blood pressure; SD, standard deviation.

### 3.2 Physical fitness variables and heart rate variability

The interaction between the two factors reached statistical significance for all the analysed variables (*p* < 0.01). Intra-group comparisons in both EG showed significant improvements in all fitness-related variables (*p* ≤ 0.04), except for maximal aerobic power in the AUG. ES based on Cohen's *d* ranged from trivial to large (*d* = 0.15 to *d* = 1.12) for the AUG and from small to large (*d* = 0.23 to *d* = 1.63) for the PTG. In contrast, the CG did not show statistically significant increases in any variable. A detailed summary of the results and comparisons for all groups is presented in Table 2.

**Table 2:** Effect of exercise on fitness variables (mean ± SD).

Variable	Group	*p* inter	PRE	POST	*p*	MC (95% CI)	Cohen's *d*
Upper Body Strength (rep)	AUG	<0.001*	21.50 ± 11.47	26.67 ± 11.66	<0.001*	5.17 (2.63, 7.71)	0.49
	PTG		24.61 ± 9.94	33.57 ± 12.18	<0.001*	8.96 (6.71, 11.20)	0.85
	CG		23.00 ± 8.86	22.93 ± 9.60	>0.999	−0.07 (−2.07, 1.93)	−0.01
	All		23.14 ± 9.86	27.39 ± 11.81	<0.001*	4.69 (3.82, 5.55)	0.45
Lowe Body Strength (rep)	AUG	<0.001*	47.61 ± 7.91	57.39 ± 8.10	<0.001*	9.78 (6.05, 13.51)	1.12
	PTG		50.00 ± 8.57	64.22 ± 6.82	<0.001*	14.22 (10.92, 17.52)	1.63
	CG		44.69 ± 10.13	44.79 ± 9.48	>0.999	0.10 (−2.84, 3.05)	0.01
	All		47.19 ± 9.26	54.41 ± 11.86	<0.001*	8.03 (6.77, 9.30)	0.92
VO_2_ peak (ml·kg^−1^·min^−1^)	AUG	<0.001*	33.32 ± 7.49	34.94 ± 6.49	0.037*	1.62 (0.05, 3.19)	0.21
	PTG		33.99 ± 8.17	36.80 ± 8.26	<0.001*	2.81 (1.42, 4.20)	0.37
	CG		32.21 ± 7.92	31.16 ± 7.28	0.177	−1.05 (−2.27, 0.18)	−0.14
	All		33.08 ± 7.82	33.99 ± 7.74	<0.001*	1.13 (0.59, 1.66)	0.15
Total Test Time (s)	AUG	<0.001*	533.00 ± 128.18	579.72 ± 129.09	0.037*	46.72 (1.51, 91.94)	0.34
	PTG		526.61 ± 131.46	600.17 ± 148.58	<0.001*	73.57 (33.56, 113.57)	0.54
	CG		500.38 ± 148.64	493.31 ± 129.34	>0.999	−7.07 (−42.69, 28.56)	−0.05
	All	517.39 ± 136.88	550.64 ± 142.66	<0.001*	37.74 (22.42, 53.06)	0.28
Maximal Aerobic Power (w)	AUG	<0.001*	158.99 ± 82.20	170.75 ± 82.15	0.077	11.75 (−0.62, 24.12)	0.15
	PTG		138.53 ± 54.31	156.43 ± 65.64	<0.001*	17.91 (6.96, 28.85)	0.23
	CG		206.83 ± 87.86	205.58 ± 84.19	>0.999	−1.25 (−10.99, 8.50)	−0.02
	All		172.09 ± 81.64	180.48 ± 79.97	<0.001*	9.47 (5.28, 13.66)	0.12
Ln-rMSSD (ms)	AUG	<0.001*	2.90 ± 0.69	3.21 ± 0.66	0.003*	0.31 (0.07, 0.55)	0.46
	PTG		3.09 ± 0.83	3.46 ± 0.77	<0.001*	0.37 (0.15, 0.58)	0.53
	CG		2.88 ± 0.60	2.74 ± 0.59	0.438	−0.14 (−0.33, 0.05)	−0.20
	All		2.96 ± 0.70	3.10 ± 0.73	<0.001*	0.18 (0.10, 0.26)	0.26

* Significant differences.

AUG, autonomous group; CG, control group; CI, confidence interval; Inter, interaction (time/group); Ln-rMSSD, natural logarithm root mean square of successive differences; MC, mean change; PTG, personal trainer group; rep, repetitions; SD, standard deviation; VO_2_ peak, peak oxygen uptake.

As for the ANCOVA, the results reached statistical significance for all the variables analysed (*p* < 0.01). Concretely, statistically significant differences were found for the following variables *(p* ≤ 0.01) were found between AUG and PTG change in the number of repetitions in upper and lower body strength, with the magnitude of the ES being large (*d* = –1.03 and *d* = –1.06, respectively) in favour of PTG. Furthermore, although no statistically significant differences were found, a smaller change in VO_2_ peak was observed in AUG than in PTG, with the magnitude of ES being moderate (*d* = –0.60). Comparisons between AUG and CG changes showed significant differences in favour of AUG in all variables (*p* ≤ 0.01), except for maximal aerobic power (*p* = 0.06), with moderate to large ES (*d* = 0.72 to *d* = 2.19). When comparing PTG with CG, changes were statistically superior in PTG in all variables (*p* < 0.01), with large ES (*d* = 1.07 to *d* = 3.25). A detailed summary of the comparisons between all groups is provided in Table 3.

**Table 3:** Between-group comparisons of mean change in fitness variables.

Variable	Group	*p*	*p^A^*	MD (95% CI)	Cohen's d	*p^B^*	MD (95% CI)	Cohen's d	*p^C^*	MD (95% CI)	Cohen's *d*
Upper Body Strength (rep)	AUG	<0.001*	0.005*	−3.62 (−6.30, −0.94)	−1.03	<0.001*	5.32 (2.76, 7.86)	1.51	<0.001*	8.94 (6.57, 11.31)	2.54
	PTG										
	CG										
Lower Body Strength (rep)	AUG	<0.001*	0.004*	−5.02 (−8.62, −1.43)	−1.06	<0.001*	10.39 (6.95, 13.83)	2.19	<0.001*	15.41 (12.14, 18.69)	3.25
	PTG										
	CG										
VO_2_ peak (ml·kg^−1^·min^−1^)	AUG	<0.001*	0.141	−1.25 (−2.82, 0.31)	−0.60	<0.001*	2.78 (1.29, 4.27)	1.34	<0.001*	4.03 (2.64, 5.42)	1.95
	PTG										
	CG										
Total Test Time (s)	AUG	<0.001*	0.372	−26.08 (−72.31, 20.16)	−0.43	0.007*	57.71 (13.43, 101.99)	0.94	<0.001*	83.78 (42.63, 124.94)	1.37
	PTG										
	CG										
Maximal Aerobic Power (w)	AUG	0.002*	0.527	−5.95 (−19.11, 7.21)	−0.34	0.058	12.53 (−0.35, 25.41)	0.72	0.002*	18.48 (6.02, 30.95)	1.07
	PTG										
	CG										
Ln-rMSSD (ms)	AUG	<0.001*	0.676	−0.09 (−0.33, 0.16)	−0.27	<0.001*	0.45 (0.23, 0.68)	1.43	<0.001*	0.54 (0.33, 0.76)	1.70
	PTG										
	CG										

* Significant differences.

*p*, exercise modality; *p^A^*, differences between the Autonomous Group and Personal Trainer Group; *p^B^*, differences between the Autonomous Group and Control Group; *p^C^*, differences between the Personal Trainer Group and Control Group.

AUG, autonomous group; CG, control group; Ln-rMSSD, natural logarithm root mean square of successive differences; MD, mean difference; PTG, personal trainer group; rep, repetitions; VO_2_ peak, peak oxygen uptake.

Correlation analysis showed moderate (*r* = 0.36 to *r* = 0.45) and significant (*p* < 0.01) associations between changes in HRV and changes in fitness-related variables. A detailed summary of the correlation results is provided in Table 4.

**Table 4:** Correlation of changes in HRV with improvement in physical fitness.

Variable	Pearson's *r*	*p*	95% CI
Ln-rMSSD—Upper Body Strength	0.357*	0.002	0.13, 0.55
Ln-rMSSD—Lower Body Strength	0.452*	<0.001	0.24, 0.62
Ln-rMSSD—VO_2_ peak	0.409*	<0.001	0.19, 0.59
Ln-rMSSD—Total Test Time	0.376*	0.001	0.16, 0.56
Ln-rMSSD—Maximal Aerobic Powe	0.363*	0.002	0.14, 0.55

* Significant correlation.

CI, confidence interval.

### 3.3 Adherence to physical exercise programme

No statistical differences were found between AUG and PTG in terms of total number of sessions, number of high-intensity sessions, number of low-intensity sessions or training frequency (*p* ≥ 0.25). (Supplementary Table S4).

However, in the high-intensity sessions, statistically significant differences were found between the experimental groups in the time spent within the low- and high-intensity zones (*p* < 0.01). In the AUG, a higher percentage of time was observed within the low intensity zone. In contrast, in the PTG, the percentage of time was higher in the high intensity zone. Data analysis showed a small ES magnitude in the moderate intensity zone (*d* = 0.23) and large ones in the low and high intensity zones (*d* = 1.84 and *d* = 3.07, respectively). In the low-intensity sessions, statistically significant differences were found between the experimental groups in time spent, within the zones rated as low, moderate and high intensity (*p* < 0.01). In the AUG, a higher percentage of time was observed in the low intensity zone, with the magnitude of the ES being large (*d* = 1.42). In the PTG group, the percentage of time was higher in the moderate and high intensity zone, with large ES (*d* = 1.30 and *d* = 0.97, respectively). The results of these comparisons are shown in Table 5.

**Table 5:** Comparison time in each intensity zone during sessions (mean ± SD).

Variable	Group	*n*	Descriptive	*p*	MD (95% CI)	Cohen's *d*
High Intensity Session						
T 0%–59% HR max (%)	AUG	18	55.28 ± 23.49	<0.001*	31.67 (20.59, 42.74)	1.84
	PTG	22	23.61 ± 9.48			
T 60%–79% HR max (%)	AUG	18	41.00 ± 20.82	0.479	3.41 (−6.25, 13.07)	0.23
	PTG	22	37.59 ± 7.53			
T 80%–100% HR max (%)	AUG	18	3.72 ± 3.89	<0.001*	−35.08 (−42.44, −27.72)	−3.07
	PTG	22	38.80 ± 14.99			
Low Intensity Session						
T 0%–59% HR max (%)	AUG	18	82.25 ± 17.22	<0.001*	31.95 (17.45, 46.46)	1.42
	PTG	22	50.30 ± 26.08			
T 60%–79% HR max (%)	AUG	18	17.34 ± 16.49	<0.001*	−24.83 (−37.15, −12.51)	−1.30
	PTG	22	42.17 ± 21.05			
T 80%–100% HR max (%)	AUG	18	0.41 ± 1.27	0.004*	−7.12 (−11.87, −2.37)	−0.97
	PTG	22	7.54 ± 9.87			

* Significant differences.

AUG, autonomous group; CI, confidence interval; HR max; Maximum heart rate; MD, mean difference; *n*, sample size; PTG, personal trainer group; SD, standard deviation; T, time.

### 3.4 SF-36 health survey

The interaction between the two factors reached statistical significance for the dimensions of physical role, bodily pain, vitality, and health evolution (*p* ≤ 0.03). In the AUG, only the health evolution dimension of the SF-36 questionnaire showed a significant increase after the intervention (*p* < 0.01), with a trivial to large ES magnitude (*d* = 0.17 to *d* = 1.60). In PTG, significant improvements were identified in physical role, bodily pain, general health, vitality, mental health, and health evolution at the end of the training programme (*p* ≤ 0.03), with the magnitude of ES being between small and large (*d* = 0.35 to *d* = 1.76). No significant changes were found in CG. Regardless of the group, the results showed a statistically significant increase in the physical function, general health, social function, emotional role, and mental health (*p* ≤ 0.03), with ES ranging from *d* = 0.23 to *d* = 1.25 (Supplementary Table S5).

Between-group change differences did not reach statistical significance for physical and social functions (*p* > 0.05), so pairwise comparisons were not performed. In the remaining dimensions, statistically significant differences were found between AUG and PTG change only in the vitality dimension (*p* = 0.02), with a large ES magnitude (*d* = –0.86) in favour of PTG. Comparisons between AUG and CG changes showed significant differences in favour of AUG in vitality, emotional role, and health evolution (*p* ≤ 0.04), showing small to large ES magnitudes (*d* = 0.28 to *d* = 1.31). Finally, when comparing PTG with CG, changes were statistically superior in PTG in all quality of life dimensions (*p* ≤ 0.04), showing magnitudes of ES between small and large (*d* = 0.47 to *d* = 1.93). Full details are provided in Supplementary Table S6.

### 3.5 Psychological vitality and affective state

The interaction between the two factors reached statistical significance for psychological vitality and negative affective state (*p* ≤ 0.04). In the AUG, significant improvements were observed in psychological vitality and negative affective state (*p* ≤ 0.02), showing moderate ES magnitudes for positive affective state (*d* = 0.62) and large for vitality (*d* = 0.89) and negative affective state (*d* = 0.81). In the PTG, all variables improved significantly (*p* ≤ 0.03), with ES magnitudes being moderate for positive affective state (*d* = 0.62) and large for vitality (*d* = 0.81) and negative affective state (*d* = 1.00). In contrast, CG showed no significant changes. Regardless of the group, the results showed a statistically significant increase in the psychological vitality and negative affective state (*p* ≤ 0.04), with ES the *d* = 0.66 and *d* = –0.59, respectively (Supplementary Table S7).

The overall ANCOVA reached statistical significance for all variables analysed (*p* < 0.01). Specifically, no statistically significant differences were found in the change between AUG and PTG in any variable (*p* ≥ 0.99). Comparisons between AUG and CG changes showed significant differences in favour of AUG in vitality, positive affective state and negative affective state (*p* ≤ 0.02), showing large ES magnitudes (*d* = 0.82 to *d* = 1.09). Similarly, when comparing PTG with CG, changes were statistically greater in PTG on these same variables (*p* ≤ 0.01), with ES magnitudes being large (*d* = 0.83 to *d* = 1.21). Full results are presented in Supplementary Table S8.

### 3.6 Training load

When comparing the groups, statistically significant differences (*p* < 0.01) were found between the AUG and PTG in cumulative training load in all zones in favour of the PTG, except in zone 2 (*p* = 0.24). The magnitude of the ES varied between small for zone 2 (*d* = 0.38) and large for the rest of the zones and for the total training load (*d* = 1.27 to *d* = 2.79). Detailed results of these comparisons are presented in Table 6.

**Table 6:** Comparison of the training load during the programme (mean ± SD).

Variable	Group	*n*	Descriptive	*p*	MD (95% CI)	Cohen's *d*
eTRIMP Z1 (a.u.)	AUG	18	44,057.94 ± 19,357.87	<0.001*	19,541.40 (9,626.80, 2,94,566.00)	1.27
	PTG	22	24,516.55 ± 11,240.29			
eTRIMP Z2 (a.u.)	AUG	18	26,190.44 ± 13,819.55	0.237	−4,030.83 (−10,829.11, 2,767.45)	−0.38
	PTG	22	30,221.27 ± 6,886.46			
eTRIMP Z3 (a.u.)	AUG	18	17,967.83 ± 17,700.58	<0.001*	−20,936.58 (−30,352.69, −11,520.46)	−1.43
	PTG	22	38,904.41 ± 11,573.24			
eTRIMP Z4 (a.u.)	AUG	18	6,301.11 ± 7,572.89	<0.001*	−41,010.71 (−50,481.51, −31,539.90)	−2.79
	PTG	22	47,311.82 ± 18,592.00			
eTRIMP Z5 (a.u.)	AUG	18	422.78 ± 882.42	<0.001*	−34,043.13 (−47,748.29, −20,337.97)	−1.60
	PTG	22	34,465.91 ± 28,643.23			
eTRIMP T (a.u.)	AUG	18	94,940.11 ± 40,433.98	<0.001*	−80,479.84 (−1,09,654.63, −51,305.05)	−1.78
	PTG	22	1,75,419.96 ± 48,961.2			
sRPE T (a.u.)	AUG	18	3,46,307.83 ± 1,68,293.42	0.844	11,106.52 (−1,02,477.73, 1,24,690.77)	0.06
	PTG	22	3,35,201.32 ± 1,82,941.95			

* Significant differences.

a.u., arbitrary units; AUG, autonomous group; CI, confidence interval; eTRIMP, edwards training impulse; MD, mean difference; *n*, sample size; PTG, personal trainer group; SD, standard deviation; sRPE, session rating of perceived exertion; T, total; Z, zone.

### 3.7 Adverse events

No adverse events directly related to the interventions were reported. Prior to the study, some participants faced technical problems or schedule changes. During the intervention, 14 participants dropped out due to work, family or academic commitments, health problems (such as minor surgeries or COVID-19) and holidays.

## 4 Discussion

The aim of this study was to evaluate adherence and the effects of Autonomous Training (AUG) via the Selftraining UMH app and face-to-face Personal Trainer-Led (PTG) training group, both guided by HRV, in sedentary adults. Both intervention groups showed significant improvements in upper and lower body strength, aerobic capacity (VO_2_ peak), total test time, and HRV. However, only the PTG achieved statistically significant improvements in maximal aerobic power. Adherence was similar between groups, but the PTG demonstrated greater consistency in session intensity and frequency of high-intensity activities.

In between-group comparisons, statistically significant differences in upper and lower body strength were found when comparing AUG with PTG. In addition, both intervention groups showed significant differences in strength, aerobic capacity and HRV compared to the CG. However, improvements in VO_2_ peak reached statistical significance only when comparing PTG with CG.

### 4.1 Selftraining UMH mobile app

Our results regarding the impact of the Selftraining UMH app on improving aerobic capacity (MD = 1.62 ml·kg^−1^·min^−1^ intra-group and MD = 2.78 ml·kg^−1^·min^−1^ vs. CG) are consistent with previous studies on exercise apps. For example, App-tivate improved VO_2_ peak by 1.4 ml·kg^−1^·min^−1^ intra-group and 2.0 ml·kg^−1^·min^−1^ vs. controls in people with metabolic disorders (57). The Harufit app, in patients recovering from acute myocardial infarction, increased VO_2_ peak by 3.18 ml·kg^−1^·min^−1^ in the intra-group comparisons (58). In young people, Clinic C, integrated with virtual reality and exercise bikes, also achieved an increase of 3.18 ml·kg^−1^·min^−1^ (59). Other apps, such as Strava, Pacer, MapMyWalk, and Pokémon Go, used for step monitoring in adolescents, reported increases that ranged from 1.18 and MD = 0.95 ml·kg^−1^·min^−1^ in intra-group analyses to 0.57 and 0.43 ml·kg^−1^·min^−1^ compared to CGs, although without structured programmes (60). In adults, Acupedo (walking), FMTK (bodyweight exercises at home), and LogMyFood (diet tracking via photographic records) reported improvements of up to 1.32 ml·kg^−1^·min^−1^ in six weeks and 1.07 ml·kg^−1^·min^−1^ at 12 weeks in intra-group analyses. Previous reviews support these results, highlighting significant improvements in different populations using apps that track daily steps (61, 62). Mobile apps can, therefore, achieve comparable physical adaptations to supervised programmes, provided that exercise prescriptions are equivalent (63) and adherence is adequate (64). Furthermore, a systematic review with meta-analysis suggests that the impact on VO_2_ peak is greater when mobile apps include features such as continuous monitoring (65) and automated feedback (66). Notably, improvements in aerobic capacity tend to be more consistent in short-term studies lasting less than 12 weeks (67).

The increased strength in both upper and lower body PTG (*p* < 0.01) can be attributed to technical adjustments by the trainer, which optimise muscle activation and motor unit recruitment. During the first 6–8 weeks of training, strength improvements are predominantly neurological, whereas in later weeks (12–26), they are due to myofibrillar hypertrophy and possible transitions between fast muscle fibres types IIa and IIx (68). Additionally, the higher workload in high-intensity zones (80%–100% HR max) in the PTG contributed to significant differences with respect to the AUG (69). Results on the use of mobile apps for strength development are varied and depend on the programme design and population. A recent study showed significant improvements in upper and lower body strength in active (*p* = 0.02 and *p* = 0.02, respectively) and inactive (*p* = 0.02 and *p* < 0.01, respectively) adolescents in the within-group analysis (60). However, only the active subjects showed differences in the lower body compared to the CG (*p* = 0.03), possibly due to their regular physical activity (60). In young adults (59), improvements in lower body strength (*p* = 0.01) were observed after using mobile technologies, but these were smaller compared to traditional interventions without the use of technology or supervised HIIT (70–73).

The PTG was the only one to show a significant improvement in maximal aerobic power (*p* < 0.01). The effect of training guided by heart rate variability in a supervised manner for the improvement of maximal power has been previously demonstrated in studies carried out with different populations (46, 48). However, although the change did not reach statistical significance in this study, we found an improvement in the AUG of power of a magnitude of 11.75 watts. Furthermore, our results show that the groups did not have the same power values before training, and when the changes are analysed, taking into account the values before the intervention, no differences are found in the improvement in this variable (*p* = 0.53).

These greater improvements may be attributed to greater exposure to high-intensity zones during training sessions. Evidence suggests that individuals participating in self-contained exercise programmes often do not reach the prescribed intensity during high-intensity sessions (74–78), despite completing the same total number of sessions, including both high- and low-intensity sessions. This difference is likely to be influenced by psychological, social and technical factors.

The improvements observed in both modalities are due to the use of periodisation techniques, which structure loading and recovery cycles. In addition, the manipulation of FITT principles (frequency, intensity, time, type) allowed for personalised adjustments, avoiding plateaus and injuries (79–81). The inclusion of HIIT, a time-efficient method, and resistance exercises favoured overall improvements in physical performance, mobility and functional independence, essential for quality of life (82, 83). This comprehensive approach maximises exercise adherence and ensures sustainable benefits (84).

### 4.2 HRV-based physical exercise programmes

The results are consistent with previous research that has demonstrated the efficacy of HRV-guided exercise programmes in optimising physical performance (85) and reducing the risk of overtraining (86). This approach allows for daily adjustments in exercise load according to individual physiological state. Previous studies have reported significant improvements in fitness parameters in diverse populations, including sedentary (87), recreational runners (49, 88), elite athletes (46, 48, 89) and patients with pathologies (90). In particular, HRV-guided training is superior to predefined programmes as it personalises the load to maximise physiological adaptations and minimise variability in individual responses (17, 91, 92).

In our study, both AUG and PTG showed increases in rMSSD, with mean differences (MD) of 0.45 milliseconds and 0.54 milliseconds, respectively, vs. CG. This highlights the positive effect of exercise on parasympathetic nervous system (PNS) modulation. Consistent with this, previous research has reported significant improvements in rMSSD in healthy sedentary people (93) and patients with various pathologies (94–96). Additionally, significant intra-group improvements were observed in our study, with pre and post-intervention MD increases of 0.31 milliseconds in the AUG and 0.37 milliseconds in the PTG. Similar findings were reported by Picard et al. (96), who observed a standard MD increase of 0.30 milliseconds in rMSSD in within-group analyses. The observed improvements reinforce the idea that physical exercise may be an effective non-pharmacological strategy to improve cardiac vagal modulation and reduce mortality risk (97–99).

Unlike other applications, the Selftraining UMH app integrates an HRV-based programme that measures, monitors exercise responses and prescribes daily routines tailored to individual needs. In addition, HRV can be monitored during activity to ensure that the target intensity is reached.

### 4.3 Adherence

Although there was no significant difference in the total number of sessions completed between AUG and PTG (MD = 23.28 vs. MD = 24.52), nor in number of low-intensity (MD = 9.56 vs. MD = 9.96) and high-intensity sessions (MD = 13.72 vs. MD = 14.57), the AUG adherence rate (70.56%) was notably higher than the average for online interventions (≈50%) (100). This can be attributed to the effective use of new technologies and the flexibility of the programme, which allowed participants to adjust the duration and frequency of sessions according to their needs, facilitating integration into their daily routines (101–104). Personalisation within a structured framework not only favours engagement but also the long-term sustainability of the programme.

Understanding the factors that influence adherence to physical exercise is essential for ensuring long-term commitment and success (105–107). Men are generally more motivated by challenges, competition, social recognition, and the desire to increase muscle size and strength. They also tend to prefer high-intensity, competitive exercises that focus on the upper body. In contrast, women are typically driven by goals related to enhancing their physical appearance, toning their muscles, and managing body weight. Additionally, they show a greater preference for supervised activities that target the lower body (108).

Sex-based differences in adaptation to training have a physiological basis. Variations have been identified in fatigue resistance (109–111), inflammatory response following eccentric exercise (112), recovery time (113), and muscle fibre composition (114, 115). While Wilmore (116) found that both sexes achieved similar relative gains in strength and lean body mass, subsequent studies have produced mixed results (117–122), depending on whether the data are presented in absolute or relative terms. A recent meta-analysis by Roberts et al. (123) found that men and women respond similarly in terms of strength and muscle size development in the lower body. However, women demonstrate greater relative strength gains in the upper body.

During endurance training, women utilise more fatty acids than men, which enhances carbohydrate conservation and reduces neuromuscular fatigue, with fewer peripheral alterations. It has been suggested that oestrogen may have a protective effect against damage caused by repetitive eccentric muscle actions (124). At the cardiac level, functional adaptations of the left ventricle to endurance training are less pronounced or even absent in women despite similar hypertrophic responses in both sexes (125). Furthermore, oxygen transport, constrained by the lower total haemoglobin mass in women (126, 127), limits their ability to achieve a higher VO_2_ peak (128). Their relatively lower blood volume also restricts the amount of blood available to active muscles without compromising supply to other tissues, potentially impacting aerobic performance (129).

### 4.4 Quality of life and well-being

The findings of this study highlight the significant impact of structured physical exercise programmes on health-related quality of life and psychological well-being, as evidenced by the improvements observed in both the AUG and PTG compared to the CG. These results reflect the complex interactions between the physiological benefits of exercise and participants' subjective perceptions, aligning with previous research.

In the PTG, significant improvements were noted across multiple SF-36 dimensions, including physical role (*p* = 0.02), bodily pain (*p* = 0.03), general health (*p* = 0.03), vitality (*p* < 0.01), mental health (*p* = 0.01), and health evolution (*p* < 0.01). These outcomes can be attributed to the structured, supervised nature of the programme, which ensured proper intensity, adherence, and consistent feedback (130). This approach likely optimised the physical and mental health benefits of exercise by promoting regional musculoskeletal adaptations and alleviating dysfunction, as well as through the progressive manipulation of training variables (131, 132). Conversely, the AUG showed significant improvements primarily in health evolution (*p* < 0.01), with moderate effect sizes observed in vitality (*d* = 0.62), emotional role (*d* = 0.57), and mental health (*d* = 0.50). These results demonstrate the effectiveness of self-directed exercise programmes, particularly in enhancing perceived energy and reducing fatigue, though the lack of professional feedback and social interaction may limit adherence and training intensity, which could lead to negligible effects in psychological assessments. Previous research has shown that combining resistance training with aerobic exercise yields greater benefits compared to aerobic exercise alone (133). In contrast, the CG did not experience significant changes in any of the SF-36 dimensions, underscoring the importance of regular exercise, whether supervised or self-directed, in improving quality of life and psychological well-being. These findings are consistent with previous studies highlighting exercise's role in alleviating physical and emotional limitations (134).

Psychological well-being outcomes further support the benefits of exercise. Intra-group analyses revealed significant improvements in vitality (MD = 1.08) and negative affective state (MD = 0.91) for the AUG, while the PTG showed improvements in vitality (MD = 0.98), positive affective state (MD = 0.79), and negative affective state (MD = 1.11). Between-group comparisons confirmed significant differences between the intervention groups and the CG in all variables (*p* < 0.05). These results can be attributed to the capacity of exercise to reduce stress, enhance emotional regulation, and foster positive psychological states (135). While both intervention groups achieved notable gains, the PTG demonstrated slightly greater improvements, likely due to the structured feedback and motivational environment provided by trainer-led sessions.

The absence of significant changes in social and physical function may be due to a ceiling effect, as these dimensions are less impacted in moderately active populations (136). Nonetheless, the differences in changes between groups due to exercise modality underscores the importance of programme design in maximising perceived benefits. Overall, these findings reinforce the multifaceted benefits of well-designed exercise programmes on health-related quality of life and psychological well-being.

### 4.5 Training load

Training load, as measured by eTRIMP, was significantly higher in the PTG (*p* < 0.01), while the sRPE method showed no difference in the subjective perception of load between groups. This suggests that while eTRIMP reflects a greater reliance on high-intensity sessions in the trained group (facilitated by the coach's technical guidance and group training dynamics), the perceived demand and appropriateness of the exercise load were comparable between groups. In the AUG group, this equivalence can be attributed to the use of tools such as HRV monitoring and autonomous decision-making based on these readings. However, the shorter cumulative time in high-intensity zones in the AUG (3.72% vs. 38.80%, *p* < 0.01) probably explains the smaller improvements in fitness variables. These results emphasise the need to optimise feedback strategies in self-directed programmes to ensure greater exposure to high-intensity training. On the other hand, AUG exhibited a greater emphasis on low-intensity sessions, which may be more appropriate for individuals with initial fitness limitations but less effective in driving significant physiological adaptations. Previous evidence indicates that self-selected intensity in exercise is often inferior to supervised sessions in promoting improvements in fitness (74–78), probably due to psychological, social and technical factors. Although the total tolerated load was equivalent between groups, the lack of differences in perceived load, despite substantial variations in intensity distribution, suggests that the presence of a coach may not be essential to induce adaptations in sedentary populations. In self-directed programmes, HRV emerges as a critical tool to guide decision-making and replicate the technical adjustments typically associated with direct supervision.

Group interaction in supervised programmes also plays a crucial role, enhancing performance through implicit competition, social pressure and motivational support (137–140). According to self-determination theory (141), group environments that promote a positive motivational climate reinforce connectedness, competence and autonomy, facilitating more intense and sustained effort over time.

### 4.6 Limitations

This study had several limitations. First, the final sample number of participants who completed the study in both experimental groups reduced statistical power, thereby limiting the applicability of the findings to the broader population. Second, the proportion of women was twice that of men. The low participation of men in structured physical exercise programmes may be influenced by a combination of psychological, social and cultural factors. Gender stereotypes play a significant role, as men often perceive such programmes as less appealing, associating them with lower intensity compared to activities like weightlifting (142). Furthermore, these programmes may not align with men's preferences for independence, competitiveness and self-direction, as they tend to prefer varying their routines according to personal interests and goals rather than adhering to a pre-defined structure (143–145). Some men also perceive these programmes as insufficiently challenging (146) or view them as socially accepted activities primarily targeted at women, which reinforces their lack of interest (147, 148). Lastly, the study did not include a post-intervention follow-up to assess whether participants who completed the programme-maintained adherence to physical exercise over time. Such follow-up data could provide valuable insights into the long-term effectiveness of the intervention.

### 4.7 Relevance for clinical practice and public health

The observed increases in VO_2_ peak 1.62 ml·kg^−1^·min^−1^ in the AUG and 2.81 ml·kg^−1^·min^−1^ in the PTG, may have significant long-term health implications. Research suggests that a 1 ml·kg^−1^·min^−1^ increase in VO_2_ peak is associated with a 9% reduction in the relative risk of all-cause mortality (149, 150), an impact comparable to a 10 cm decrease in waist circumference or a 10-mmHg reduction in systolic blood pressure (151). Importantly, aerobic capacity, like physical exercise, independently reduces mortality risk, emphasising the critical relevance of the improvements in VO_2_ peak observed in this study (152).

Technology-guided autonomous physical exercise programmes, such as the Selftraining UMH app, provide an accessible and flexible alternative, particularly in scenarios where face-to-face attendance is not feasible. While it is not intended to replace professionally supervised training, it can serve as an initial step toward online programmes, potentially encouraging participants to seek face-to-face guidance in the future. For sustained long-term benefits, a hybrid approach that combines technological autonomy with human supervision may be particularly effective (153). In this regard, the results highlight the relevance of maintaining constant user interaction with the app to maximise the intervention's success (154). This highlights the potential for mobile apps to bridge gaps in access to exercise programmes while complementing traditional supervised modalities, thereby enhancing public health outcomes.

### 4.8 Practical applications

For older adults, the application includes a library of video-based training sessions specifically designed for their age group. The updated version of the application, “Selftraining Health”, is aimed at enhancing emotional well-being and physical health in individuals with mental health conditions and addictions.

The Selftraining UMH application, based on HRV, could be implemented in rehabilitation programmes. Before its use for different clinical populations, it would be necessary to adapt the training sessions and validate them according to the specific needs of the target population.

The practical implementation of this application in digital health training enables users, regardless of their familiarity with technology, to access personalised exercise programmes that can be performed independently. Moreover, by integrating wearable technologies to track HRV, Selftraining UMH facilitates precise monitoring of the user's physical condition, promoting continuous education in the use of emerging health technologies. This combination of physical exercise and digital training significantly contributes to improving public health and quality of life while fostering digital literacy in the healthcare domain.

### 4.9 Future research

Future research involving the Selftraining UMH application could adopt a crossover design, allowing participants in the Self-Directed Training modality to switch to the Personal Trainer-Led Training modality and vice versa. This approach would enable a direct comparison of the results obtained across both training modalities. Additionally, future studies could explore the effectiveness of the programme in other age groups, such as younger adults and older adults, to evaluate its applicability and impact across a broader demographic spectrum.

## 5 Conclusions

In conclusion, the findings of this study demonstrated comparable levels of adherence and physical fitness in both experimental groups before and after the training period. Additionally, significant improvements were observed within both groups in fitness-related variables when comparing pre- and post-intervention assessments. When analysing changes between groups, both experimental groups showed significant differences compared to the CG in fitness-related variables. However, statistical differences between the AUG and the PTG were observed only in upper and lower body strength. These results suggest that autonomously guided physical exercise, prescribed through HRV monitoring via the Selftraining UMH application, is a practical and effective tool for individuals seeking to initiate a physical exercise programme but face barriers to accessing training centres or supervised sessions due to cost or time constraints. However, given the results, supervised HRV-guided training is more effective than autonomous HRV-guided training for improving strength levels. Therefore, to optimise improvements in physical fitness, it is recommended that physical exercise programmes are carried out under the supervision of qualified exercise and sports professionals.

## Data Availability

The raw data supporting the conclusions of this article will be made available by the authors, without undue reservation.
