# Supplementary material for: Impact of heart rate variability-based exercise prescription: self-guided by technology and trainer-guided exercise in sedentary adults
Source: Front Sports Act Living. 2025 May 22;7:1578478. doi: 10.3389/fspor.2025.1578478 (PMC12137358; doi:10.3389/fspor.2025.1578478)
Supplement: Supplementary file 1 [file Table1.docx]

**Table 1**. Descriptive statistics of adult participants (mean ± SD).

| **Characteristics** | **AUG (*n* = 18)** | **PTG (*n* = 23)** | **CG (*n* = 29)** |
| --- | --- | --- | --- |
| Gender (male/female) | 8/10 | 6/17 | 9/20 |
| Age (years) | 40.50 ± 9.30 | 37.41 ± 12.53 | 36.89 ± 10.97 |
| Height (m) | 1.67 ± 0.09 | 1.64 ± 0.08 | 1.67 ± 0.11 |
| Weight (kg) | 71.06 ± 11.34 | 67.33 ± 11.85 | 73.08 ± 18.35 |
| BMI (kg∙m^−2^) | 25.65 ± 3.87 | 25.15 ± 4.41 | 26.23 ± 5.24 |
| SBP (mmHg)​​ | 124.05 ± 13.52 | 116.44 ± 8.75 | 117.97 ± 13.07 |
| DBP (mmHg)​​ | 78.68 ± 7.36 | 76.35 ± 7.36 | 74.97 ± 8.67 |
| HR_rest_ (bpm) | 71.32 ± 10.71 | 72.22 ± 14.37 | 70.53 ± 10.11 |
| HR_max_ (bpm) | 176.26 ± 18.45 | 180.61 ± 10.09 | 179.57 ± 13.47 |
| Ln-rMSSD (ms) | 2.89 ± 0.68 | 3.09 ± 0.83 | 2.88 ± 0.59 |

AUG, Autonomous Group; bpm, beats per minute; BMI, body mass index; CG, Control Group; DBP, diastolic blood pressure; HR_max_, maximum heart rate; HR_rest_, resting heart rate; Ln-rMSSD, natural logarithm natural logarithm root mean square of successive differences; n, sample number; PTG, Personal Trainer Group; SMP, systolic blood pressure; SD, standard deviation.
