# Supplementary material for: Impact of heart rate variability-based exercise prescription: self-guided by technology and trainer-guided exercise in sedentary adults
Source: Front Sports Act Living. 2025 May 22;7:1578478. doi: 10.3389/fspor.2025.1578478 (PMC12137358; doi:10.3389/fspor.2025.1578478)
Supplement: Supplementary file 2 [file Table2.docx]

**Table 2**. Effect of exercise on fitness variables (mean ± SD).

| **Variable** | **Group** | ***p* inter** | **PRE** | **POST** | ***p*** | **MC (95% CI)** | **Cohen's *d*** |
| --- | --- | --- | --- | --- | --- | --- | --- |
| Upper Body Strength (rep) | AUG | < 0.001* | 21.50 ± 11.47 | 26.67 ± 11.66 | < 0.001* | 5.17 (2.63, 7.71) | 0.49 |
|  | PTG |  | 24.61 ± 9.94 | 33.57 ± 12.18 | < 0.001* | 8.96 (6.71, 11.20) | 0.85 |
|  | CG |  | 23.00 ± 8.86 | 22.93 ± 9.60 | > 0.999 | -0.07 (-2.07, 1.93) | -0.01 |
|  | All |  | 23.14 ± 9.86 | 27.39 ± 11.81 | < 0.001* | 4.69 (3.82, 5.55) | 0.45 |
| Lowe Body Strength  (rep) | AUG | < 0.001* | 47.61 ± 7.91 | 57.39 ± 8.10 | < 0.001* | 9.78 (6.05, 13.51) | 1.12 |
|  | PTG |  | 50.00 ± 8.57 | 64.22 ± 6.82 | < 0.001* | 14.22 (10.92, 17.52) | 1.63 |
|  | CG |  | 44.69 ± 10.13 | 44.79 ± 9.48 | > 0.999 | 0.10 (-2.84, 3.05) | 0.01 |
|  | All |  | 47.19 ± 9.26 | 54.41 ± 11.86 | < 0.001* | 8.03 (6.77, 9.30) | 0.92 |
| VO_2_ peak  (ml·kg^-1^·min^-1^) | AUG | < 0.001* | 33.32 ± 7.49 | 34.94 ± 6.49 | 0.037* | 1.62 (0.05, 3.19) | 0.21 |
|  | PTG |  | 33.99 ± 8.17 | 36.80 ± 8.26 | < 0.001* | 2.81 (1.42, 4.20) | 0.37 |
|  | CG |  | 32.21 ± 7.92 | 31.16 ± 7.28 | 0.177 | -1.05 (-2.27, 0.18) | -0.14 |
|  | All |  | 33.08 ± 7.82 | 33.99 ± 7.74 | < 0.001* | 1.13 (0.59, 1.66) | 0.15 |
| Total Test Time (s) | AUG | < 0.001* | 533.00 ± 128.18 | 579.72 ± 129.09 | 0.037* | 46.72 (1.51, 91.94) | 0.34 |
|  | PTG |  | 526.61 ± 131.46 | 600.17 ± 148.58 | < 0.001* | 73.57 (33.56, 113.57) | 0.54 |
|  | CG |  | 500.38 ± 148.64 | 493.31 ± 129.34 | > 0.999 | -7.07 (-42.69, 28.56) | -0.05 |
|  | All |  | 517.39 ± 136.88 | 550.64 ± 142.66 | < 0.001* | 37.74 (22.42, 53.06) | 0.28 |
| Maximal Aerobic Power (w) | AUG | < 0.001* | 158.99 ± 82.20 | 170.75 ± 82.15 | 0.077 | 11.75 (-0.62, 24.12) | 0.15 |
|  | PTG |  | 138.53 ± 54.31 | 156.43 ± 65.64 | < 0.001* | 17.91 (6.96, 28.85) | 0.23 |
|  | CG |  | 206.83 ± 87.86 | 205.58 ± 84.19 | > 0.999 | -1.25 (-10.99, 8.50) | -0.02 |
|  | All |  | 172.09 ± 81.64 | 180.48 ± 79.97 | < 0.001* | 9.47 (5.28, 13.66) | 0.12 |
| Ln-rMSSD (ms) | AUG | < 0.001* | 2.90 ± 0.69 | 3.21 ± 0.66 | 0.003* | 0.31 (0.07, 0.55) | 0.46 |
|  | PTG |  | 3.09 ± 0.83 | 3.46 ± 0.77 | < 0.001* | 0.37 (0.15, 0.58) | 0.53 |
|  | CG |  | 2.88 ± 0.60 | 2.74 ± 0.59 | 0.438 | -0.14 (-0.33, 0.05) | -0.20 |
|  | All |  | 2.96 ± 0.70 | 3.10 ± 0.73 | < 0.001* | 0.18 (0.10, 0.26) | 0.26 |

AUG, Autonomous Group; CG, Control Group; CI, confidence interval; Inter, interaction (time/group); Ln-rMSSD, natural logarithm root mean square of successive differences; MC, mean change; PTG, Personal Trainer Group; rep, repetitions; SD, standard deviation; VO_2_ peak, peak oxygen uptake.

*, significant differences.
