# Supplementary material for: Impact of heart rate variability-based exercise prescription: self-guided by technology and trainer-guided exercise in sedentary adults
Source: Front Sports Act Living. 2025 May 22;7:1578478. doi: 10.3389/fspor.2025.1578478 (PMC12137358; doi:10.3389/fspor.2025.1578478)
Supplement: Supplementary file 3 [file Table3.docx]

**Table 3**. Between-group comparisons of mean change in fitness variables.

| **Variable** | **Group** | ***p*** | ***p ^A^*** | **MD (95% CI)** | **Cohen's d** | ***p ^B^*** | **MD (95% CI)** | **Cohen's d** | ***p ^C^*** | **MD (95% CI)** | **Cohen's *d*** |
| --- | --- | --- | --- | --- | --- | --- | --- | --- | --- | --- | --- |
| Upper Body Strength (rep) | AUG | < 0.001* | 0.005* | -3.62  (-6.30, -0.94) | -1.03 | < 0.001* | 5.32  (2.76, 7.86) | 1.51 | < 0.001* | 8.94  (6.57, 11.31) | 2.54 |
|  | PTG |  |  |  |  |  |  |  |  |  |  |
|  | CG |  |  |  |  |  |  |  |  |  |  |
| Lower Body Strength  (rep) | AUG | < 0.001* | 0.004* | -5.02  (-8.62, -1.43) | -1.06 | < 0.001* | 10.39  (6.95, 13.83) | 2.19 | < 0.001* | 15.41  (12.14, 18.69) | 3.25 |
|  | PTG |  |  |  |  |  |  |  |  |  |  |
|  | CG |  |  |  |  |  |  |  |  |  |  |
| VO_2_ peak  (ml·kg^-1^·min^-1^) | AUG | < 0.001* | 0.141 | -1.25  (-2.82, 0.31) | -0.60 | < 0.001* | 2.78  (1.29, 4.27) | 1.34 | < 0.001* | 4.03  (2.64, 5.42) | 1.95 |
|  | PTG |  |  |  |  |  |  |  |  |  |  |
|  | CG |  |  |  |  |  |  |  |  |  |  |
| Total Test Time (s) | AUG | < 0.001* | 0.372 | -26.08  (-72.31, 20.16) | -0.43 | 0.007* | 57.71  (13.43, 101.99) | 0.94 | < 0.001* | 83.78  (42.63, 124.94) | 1.37 |
|  | PTG |  |  |  |  |  |  |  |  |  |  |
|  | CG |  |  |  |  |  |  |  |  |  |  |
| Maximal Aerobic Power (w) | AUG | 0.002* | 0.527 | -5.95  (-19.11, 7.21) | -0.34 | 0.058 | 12.53  (-0.35, 25.41) | 0.72 | 0.002* | 18.48  (6.02, 30.95) | 1.07 |
|  | PTG |  |  |  |  |  |  |  |  |  |  |
|  | CG |  |  |  |  |  |  |  |  |  |  |
| Ln-rMSSD (ms) | AUG | < 0.001* | 0.676 | -0.09  (-0.33, 0.16) | -0.27 | < 0.001* | 0.45  (0.23, 0.68) | 1.43 | < 0.001* | 0.54  (0.33, 0.76) | 1.70 |
|  | PTG |  |  |  |  |  |  |  |  |  |  |
|  | CG |  |  |  |  |  |  |  |  |  |  |

AUG, Autonomous Group; CG, Control Group; Ln-rMSSD, natural logarithm root mean square of successive differences; MD, mean difference; PTG, Personal Trainer Group; rep, repetitions; VO_2_ peak, peak oxygen uptake.

*, significant differences. *p*, exercise modality; *p ^A^*, differences between the Autonomous Group and Personal Trainer Group; *p ^B^*, differences between the Autonomous Group and Control Group; *p ^C^*, differences between the Personal Trainer Group and Control Group.
