# Supplementary material for: Impact of heart rate variability-based exercise prescription: self-guided by technology and trainer-guided exercise in sedentary adults
Source: Front Sports Act Living. 2025 May 22;7:1578478. doi: 10.3389/fspor.2025.1578478 (PMC12137358; doi:10.3389/fspor.2025.1578478)
Supplement: Supplementary file 4 [file Table5.docx]

**Table 5**. Comparison time in each intensity zone during sessions (mean ± SD).

| **Variable** | **Group** | **n** | **Descriptive** | ***p*** | **MD (95% CI)** | **Cohen's *d*** |
| --- | --- | --- | --- | --- | --- | --- |
| **High Intensity Session** |  |  |  |  |  |  |
| T 0-59% HR max (%) | AUG | 18 | 55.28 ± 23.49 | < 0.001* | 31.67 (20.59, 42.74) | 1.84 |
|  | PTG | 22 | 23.61 ± 9.48 |  |  |  |
| T 60-79% HR max (%) | AUG | 18 | 41.00 ± 20.82 | 0.479 | 3.41 (-6.25, 13.07) | 0.23 |
|  | PTG | 22 | 37.59 ± 7.53 |  |  |  |
| T 80-100% HR max (%) | AUG | 18 | 3.72 ± 3.89 | < 0.001* | -35.08 (-42.44, -27.72) | -3.07 |
|  | PTG | 22 | 38.80 ± 14.99 |  |  |  |
| **Low Intensity Session** |  |  |  |  |  |  |
| T 0-59% HR max (%) | AUG | 18 | 82.25 ± 17.22 | < 0.001* | 31.95 (17.45, 46.46) | 1.42 |
|  | PTG | 22 | 50.30 ± 26.08 |  |  |  |
| T 60-79% HR max (%) | AUG | 18 | 17.34 ± 16.49 | < 0.001* | -24.83 (-37.15, -12.51) | -1.30 |
|  | PTG | 22 | 42.17 ± 21.05 |  |  |  |
| T 80-100% HR max (%) | AUG | 18 | 0.41 ± 1.27 | 0.004* | -7.12 (-11.87, -2.37) | -0.97 |
|  | PTG | 22 | 7.54 ± 9.87 |  |  |  |

AUG, Autonomous Group; CI, confidence interval; HR max; Maximum heart rate; MD, mean difference; n, sample size; PTG, Personal Trainer Group; SD, standard deviation; T, time.

*, significant differences.
