# Supplementary material for: Impact of heart rate variability-based exercise prescription: self-guided by technology and trainer-guided exercise in sedentary adults
Source: Front Sports Act Living. 2025 May 22;7:1578478. doi: 10.3389/fspor.2025.1578478 (PMC12137358; doi:10.3389/fspor.2025.1578478)
Supplement: Supplementary file 5 [file Table6.docx]

**Table 6.** Comparison of the training load during the programme (mean ± SD).

| **Variable** | **Group** | **n** | **Descriptive** | ***p*** | **MD (95% CI)** | **Cohen's *d*** |
| --- | --- | --- | --- | --- | --- | --- |
| eTRIMP Z1  (a.u.) | AUG | 18 | 44057.94 ± 19357.87 | < 0.001* | 19541.40 (9626.80, 294566.00) | 1.27 |
|  | PTG | 22 | 24516.55 ± 11240.29 |  |  |  |
| eTRIMP Z2  (a.u.) | AUG | 18 | 26190.44 ± 13819.55 | 0.237 | -4030.83 (-10829.11, 2767.45) | -0.38 |
|  | PTG | 22 | 30221.27 ± 6886.46 |  |  |  |
| eTRIMP Z3  (a.u.) | AUG | 18 | 17967.83 ± 17700.58 | < 0.001* | -20936.58 (-30352.69, -11520.46) | -1.43 |
|  | PTG | 22 | 38904.41 ± 11573.24 |  |  |  |
| eTRIMP Z4  (a.u.) | AUG | 18 | 6301.11 ± 7572.89 | < 0.001* | -41010.71 (-50481.51, -31539.90) | -2.79 |
|  | PTG | 22 | 47311.82 ± 18592.00 |  |  |  |
| eTRIMP Z5  (a.u.) | AUG | 18 | 422.78 ± 882.42 | < 0.001* | -34043.13 (-47748.29, -20337.97) | -1.60 |
|  | PTG | 22 | 34465.91 ± 28643.23 |  |  |  |
| eTRIMP T  (a.u.) | AUG | 18 | 94940.11 ± 40433.98 | < 0.001* | -80479.84 (-109654.63, -51305.05) | -1.78 |
|  | PTG | 22 | 175419.96 ± 48961.2 |  |  |  |
| sRPE T  (a.u.) | AUG | 18 | 346307.83 ± 168293.42 | 0.844 | 11106.52 (-102477.73, 124690.77) | 0.06 |
|  | PTG | 22 | 335201.32 ± 182941.95 |  |  |  |

a.u., arbitrary units; AUG, Autonomous Group; CI, confidence interval; eTRIMP, Edwards Training Impulse; MD, mean difference; n, sample size; PTG, Personal Trainer Group; SD, standard deviation; sRPE, session Rating of Perceived Exertion; T, total; Z, zone.

*, significant differences.
