# Supplementary material for: Impact of heart rate variability-based exercise prescription: self-guided by technology and trainer-guided exercise in sedentary adults
Source: Front Sports Act Living. 2025 May 22;7:1578478. doi: 10.3389/fspor.2025.1578478 (PMC12137358; doi:10.3389/fspor.2025.1578478)
Supplement: Supplementary file 6 [file Datasheet1.docx]

Supplementary Material

# Definition of the levels of the training programme

## Supplementary Figure S1. Exercise session design level 1. AR, active rest; C-DOWN, cool-down; Exe, exercise; HR max, maximum heart rate; min, minutes; Rep, repetitions; s, seconds; W-UP, warm-up; W-UP SP, warm-up specific.


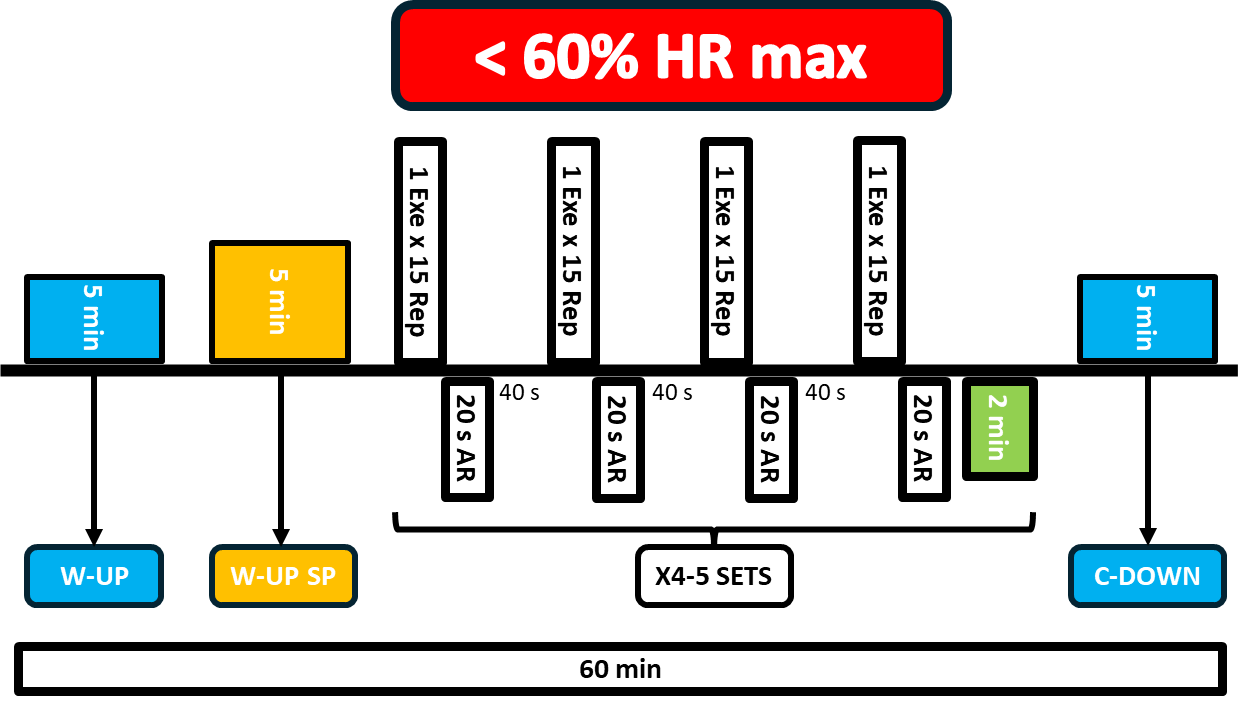


## Supplementary Figure S2. Exercise session design level 2. C-DOWN, cool-down; min, minutes; s, seconds; W-UP, warm-up; W-UP SP, warm-up specific.


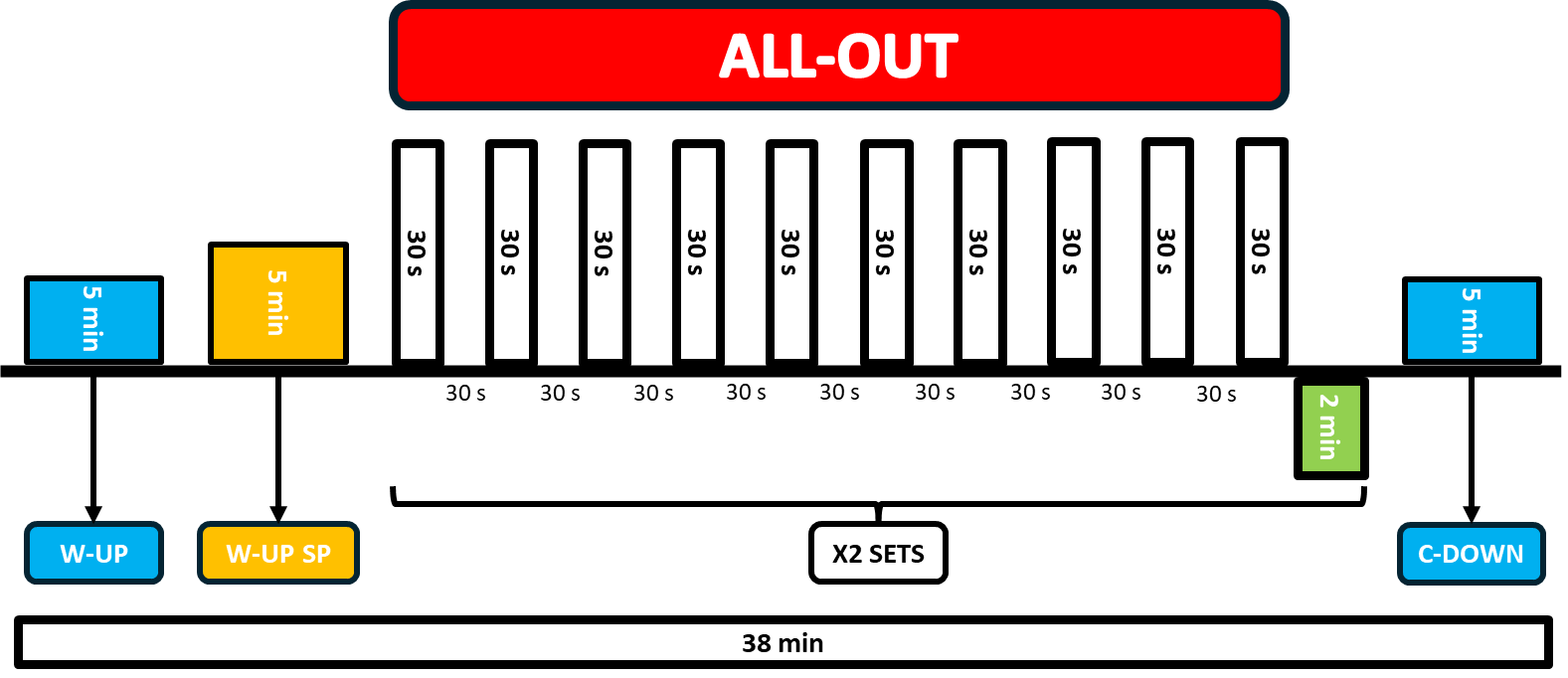


## Supplementary Figure S3. Exercise session design level 3. C-DOWN, cool-down; min, minutes; s, seconds; W-UP, warm-up; W-UP SP, warm-up specific.

**
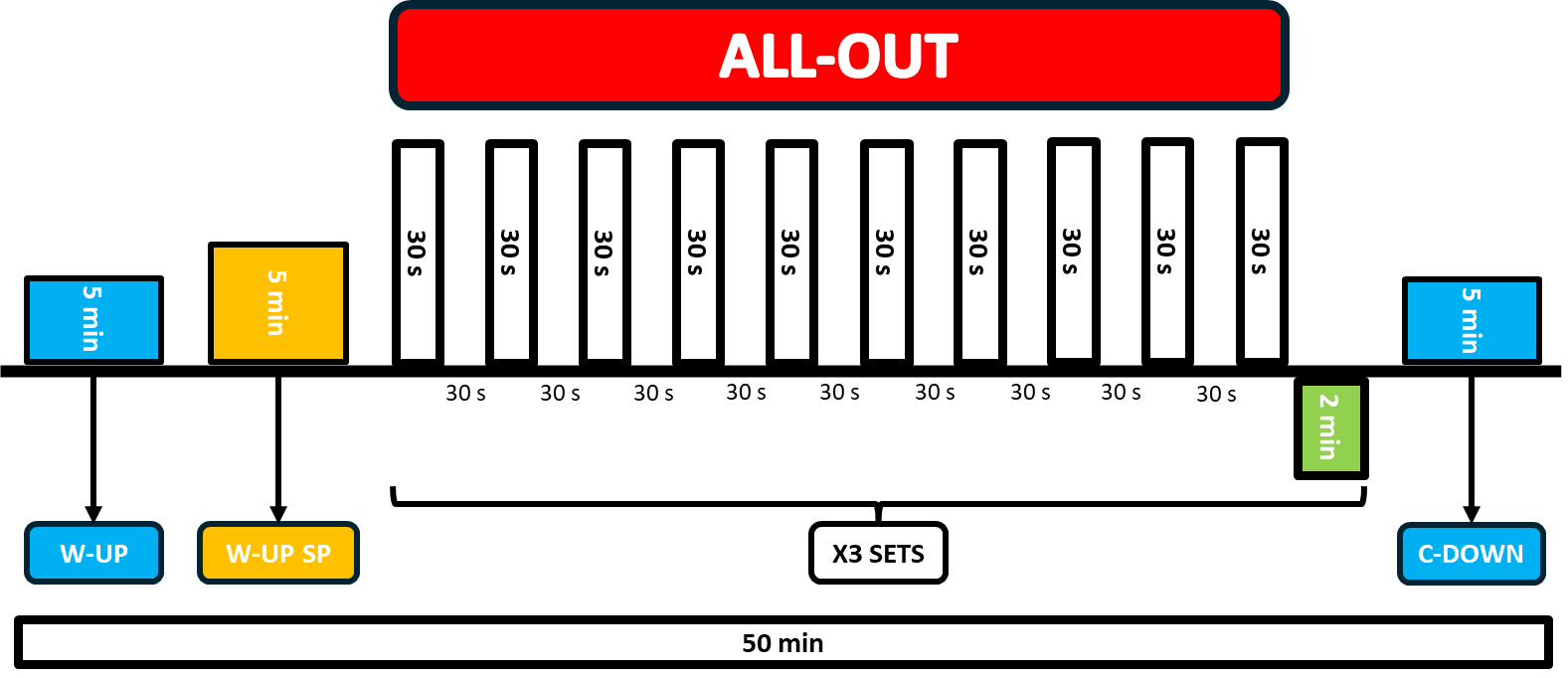
**

## Supplementary Figure S4. Exercise session design level 4. C-DOWN, cool-down; min, minutes; s, seconds; W-UP, warm-up; W-UP SP, warm-up specific.

**
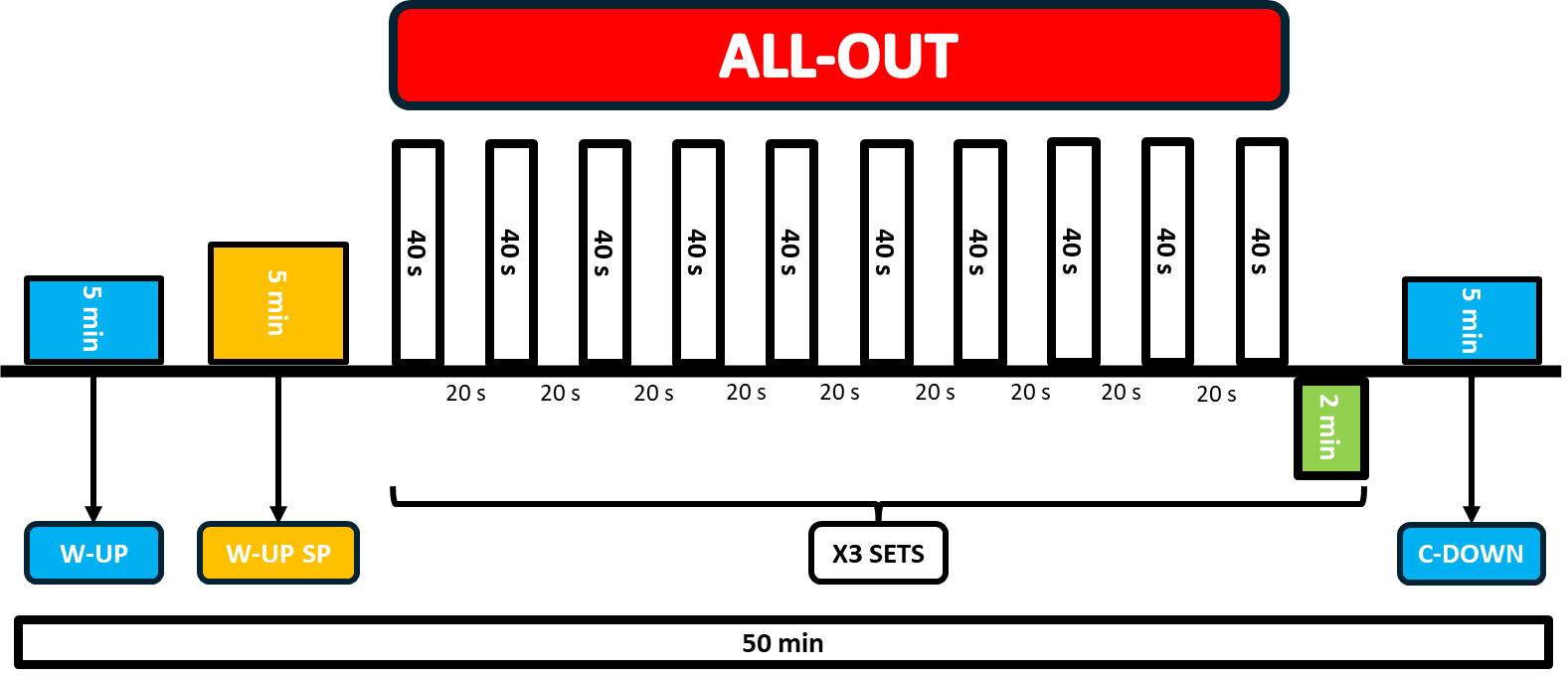
**

# Links to explanatory videos on how the Selftraining UMH app works

To ensure proper use of the “Selftraining UMH” app, several instructional videos were created, covering key aspects of the app's functionality: how to download the app (<https://youtu.be/XTIeA169DnY>), how to use the app (<https://youtu.be/T3uERGCcNbg>), how to measure HRV using the Selftraining UMH with Welltory (<https://youtu.be/WUc8lsc-VE0>), how to measure HRV with a chest strap and HR monitor via the app (<https://youtu.be/xTNF6HIU_D8>), and how to train with the app (<https://youtu.be/cjskOorjhPA>).

# Tables.

## Supplementary Table S1. Overview of the physical exercise programme for sessions.

| **Low Intensity** | | | | | | | | | |
| --- | --- | --- | --- | --- | --- | --- | --- | --- | --- |
| **Mesocycle** | **Level Session** | **Sessions/**  **Week** | **Bouts (exercises)/ Sets** | **Repetitions/ Set** | **Sets/**  **Session** | **Rest duration be active/passive (s)** | **Rest duration (min)** | **Session intensity (%HR max)** | **Total duration (min)** |
| Familiarization  (Weeks 1–2) | 1 | 3 | 4-5 | 15 | 4-5 | 20/40 | 2 | < 60 | 60 |
|  | 1 | 2 | 4-5 | 15 | 4-5 | 20/40 | 2 | < 60 | 60 |
| Mesocycle 1  (Weeks 3–5) | 1 | 1 | 4-5 | 15 | 4-5 | 20/40 | 2 | < 60 | 60 |
| Mesocycle 2  (Weeks 6–8) | 1 | 1 | 4-5 | 15 | 4-5 | 20/40 | 2 | < 60 | 60 |
| Mesocycle 3  (Weeks 9–11) | 1 | 1 | 4-5 | 15 | 4-5 | 20/40 | 2 | < 60 | 60 |
| **High-Intensity Interval Training** | | | | | | | | | |
| **Mesocycle** | **Level Session** | **Session/ Week** | **Bouts (exercises)/ Sets** | **Bouts work/Rest (s)** | **Sets/ Session** | **Work duration (min)** | **Rest duration (min)** | **Session intensity** **(%HR max)** | **Total duration (min)** |
| Familiarization  (Weeks 1–2) | 1 | 0 | - | - | - | - | - | - | - |
|  | 2 | 1 | 10-12 | 30/30 | 2 | 20-24 | 2 | All-Out | 38-40 |
| Mesocycle 1  (Weeks 3–5) | 2 | 2 | 10-12 | 30/30 | 2 | 20-24 | 2 | All-Out | 38-40 |
| Mesocycle 2  (Weeks 6–8) | 3 | 2 | 8-10 | 30/30 | 3 | 24-30 | 2 | All-Out | 44-50 |
| Mesocycle 3  (Weeks 9–11) | 4 | 2 | 8-10 | 40/20 | 3 | 24-30 | 2 | All-Out | 44-50 |

min, minutes; s, seconds; %HR max, percentage of maximum heart rate

## Supplementary Table S2. Progression of the Intensity Levels during the Training Programme.

| **Week** | **Level** | **Day 1** | **Day 2** | **Day 3** |
| --- | --- | --- | --- | --- |
| 1 | 1 | LI--L1 | LI--L1 | LI--L1 |
| 2 | 1/2 | LI--L1 | HI--L2 | LI--L1 |
| 3 | 2 | HI--L2 | HI--L2 | LI--L1 |
| 4 | 2 | HI--L2 | HI--L2 | LI--L1 |
| 5 | 2 | HI--L2 | HI--L2 | LI--L1 |
| 6 | 3 | HI--L3 | HI--L3 | LI--L1 |
| 7 | 3 | HI--L3 | HI--L3 | LI--L1 |
| 8 | 3 | HI--L3 | HI--L3 | LI--L1 |
| 9 | 4 | HI--L4 | HI--L4 | LI--L1 |
| 10 | 4 | HI--L4 | HI--L4 | LI--L1 |
| 11 | 4 | HI--L4 | HI--L4 | LI--L1 |

HI, high intensity; L, level; LI, low intensity.

## Supplementary Table S3. Possible progressions of intensity levels in the HRV-guided model.

|  | **Week 6** | | | **Week 7** | | | **Week 8** | | | **Week 9** | | |
| --- | --- | --- | --- | --- | --- | --- | --- | --- | --- | --- | --- | --- |
| **Example** | **Day 1** | **Day 2** | **Day 3** | **Day 1** | **Day 2** | **Day 3** | **Day 1** | **Day 2** | **Day 3** | **Day 1** | **Day 2** | **Day 3** |
| 4 | L3_HRV+_ | L3_HRV+_ | L1_HRV+/-_ | L3_HRV+_ | L3_HRV+_ | L1_HRV+/-_ | L1_HRV-_ | L1_HRV-_ | L1_HRV-_ | L3_HRV+_ | L3_HRV+_ | L1_HRV+/-_ |
| 3 |  |  |  |  |  |  | L1_HRV-_ | L1_HRV-_ | L3_HRV+_ | L3_HRV+_ | L1_HRV+/-_ | L3_HRV+_ |
| 2 |  |  |  |  |  |  | L3_HRV+_ | L1_HRV-_ | L3_HRV+_ | L4_HRV+_ | L1_HRV+/-_ | L4_HRV+_ |
| 1 |  |  |  |  |  |  | L3_HRV+_ | L3_HRV+_ | L1_HRV+/-_ | L4_HRV+_ | L4_HRV+_ | L1_HRV+/-_ |

HRV+, heart rate variability is within the normal range; HRV-, heart rate variability is outside the normal range; HRV+/-, regardless of the value of heart rate variability; L1, low intensity session level 1; L3, high intensity session level 3; L4, high intensity session level 4.

Table S3 discusses the training progression from week six to week nine, focusing on the dynamics of intensity level 3 and presenting four examples of progression. These examples illustrate the different possibilities that can manifest themselves throughout the training programme, considering HRV as a fundamental criterion for decision making and adjustment of sessions according to physiological state.

## Supplementary Table S4. Comparison adherence to the training programme measured as attendance and completed sessions (mean ± SD).

| **Variable** | **Group** | **n** | **Descriptive** | ***p*** | **MD (95% CI)** | **Cohen's *d*** |
| --- | --- | --- | --- | --- | --- | --- |
| TNS | AUG | 18 | 23.28 ± 9.06 | 0.579 | -1.24  (-5.74, 3.25) | -0.18 |
|  | PTG | 23 | 24.52 ± 4.99 |  |  |  |
| NLIS | AUG | 18 | 9.56 ± 4.26 | 0.698 | -0.40  (-2.48, 1.68) | -0.12 |
|  | PTG | 23 | 9.96 ± 2.21 |  |  |  |
| NHIS | AUG | 18 | 13.72 ± 6.74 | 0.609 | -0.84  (-4.15, 2.47) | -0.16 |
|  | PTG | 23 | 14.57 ± 3.58 |  |  |  |
| TF (%) | AUG | 18 | 70.56 ± 27.58 | 0.247 | -8.10  (-22.04, 5.85) | -0.37 |
|  | PTG | 23 | 78.65 ± 16.21 |  |  |  |

AUG, Autonomous Group; CI, confidence interval; MD, mean difference; n, sample size; NHIS, Number high intensity sessions; NLIS, Number low intensity sessions; PTG, Personal Trainer Group; SD, standard deviation; TF, training frequency; TNS, Total number sessions.

*, significant differences.

## Supplementary Table S5. Effect of exercise on SF-36 questionnaire (mean ± SD).

| **Dimension** | **Group** | ***p* inter** | **PRE** | **POST** | ***p*** | **MC (95% CI)** | **Cohen's *d*** |
| --- | --- | --- | --- | --- | --- | --- | --- |
| Physical  Function  (%) | AUG | 0.401 | 94.17 ± 6.47 | 96.67 ± 4.20 | > 0.999 | 2.50 (-2.61, 7.61) | 0.31 |
|  | PTG |  | 94.65 ± 6.96 | 97.17 ± 5.40 | 0.921 | 2.83 (-1.70, 7.35) | 0.35 |
|  | CG |  | 91.21 ± 10.66 | 91.55 ± 10.36 | > 0.999 | 0.35 (-3.68, 4.37) | 0.04 |
|  | All |  | 93.00 ± 8.61 | 94.71 ± 8.03 | 0.033* | 1.89 (0.16, 3.62) | 0.23 |
| Physical Role  (%) | AUG | 0.034* | 83.06 ± 20.52 | 94.44 ± 10.70 | 0.885 | 11.39 (-6.66, 29.44) | 0.41 |
|  | PTG |  | 81.52 ± 36.32 | 98.91 ± 5.21 | 0.022* | 17.39 (1.43, 33.36) | 0.63 |
|  | CG |  | 83.62 ± 31.54 | 82.76 ± 35.42 | > 0.999 | -0.86 (-15.08, 13.36) | -0.03 |
|  | All |  | 82.79 ± 30.46 | 91.07 ± 24.46 | 0.003* | 9.31 (3.19, 15.42) | 0.34 |
| Bodily  Pain  (%) | AUG | 0.023* | 79.33 ± 18.21 | 82.83 ± 18.25 | > 0.999 | 3.50 (-12.43, 19.43) | 0.17 |
|  | PTG |  | 67.83 ± 21.41 | 82.78 ± 18.35 | 0.029* | 14.96 (0.87, 29.05) | 0.71 |
|  | CG |  | 73.52 ± 21.39 | 71.00 ± 25.83 | > 0.999 | -2.52 (-15.07, 10.03) | -0.12 |
|  | All |  | 73.14 ± 20.81 | 77.91 ± 22.24 | 0.054 | 5.31 (-0.08, 10.71) | 0.25 |
| General Health  (%) | AUG | 0.115 | 66.72 ± 16.04 | 70.78 ± 15.79 | > 0.999 | 4.06 (-5.94, 14.05) | 0.23 |
|  | PTG |  | 68.65 ± 19.23 | 77.91 ± 13.08 | 0.032* | 9.26 (0.42, 18.10) | 0.53 |
|  | CG |  | 63.62 ± 20.37 | 64.69 ± 17.40 | > 0.999 | 1.07 (-6.80, 8.94) | 0.06 |
|  | All |  | 66.07 ± 18.83 | 70.60 ± 16.47 | 0.006* | 4.80 (1.41, 8.18) | 0.28 |
| Vitality  (%) | AUG | < 0.001* | 53.61 ± 18.46 | 63.33 ± 16.45 | 0.104 | 9.72 (-0.90, 20.34) | 0.62 |
|  | PTG |  | 53.91 ± 20.61 | 72.83 ± 7.66 | < 0.001* | 18.91 (9.52, 28.31) | 1.20 |
|  | CG |  | 48.79 ± 14.86 | 49.66 ± 14.51 | > 0.999 | 0.86 (-7.50, 9.23) | 0.06 |
|  | All |  | 51.71 ± 17.75 | 60.79 ± 16.52 | < 0.001* | 9.83 (6.23, 13.43) | 0.63 |
| Social Function  (%) | AUG | 0.178 | 83.61 ± 12.79 | 91.06 ± 17.52 | > 0.999 | 7.44 (-7.89, 22.78) | 0.40 |
|  | PTG |  | 81.09 ± 30.05 | 93.48 ± 11.22 | 0.106 | 12.39 (-1.18, 25.96) | 0.67 |
|  | CG |  | 86.38 ± 13.87 | 87.62 ± 18.52 | > 0.999 | 1.24 (-10.84, 13.32) | 0.07 |
|  | All |  | 83.93 ± 20.29 | 90.43 ± 16.19 | 0.009* | 7.03 (1.83, 12.22) | 0.38 |
| Emotional Role  (%) | AUG | 0.112 | 74.11 ± 38.88 | 92.61 ± 18.29 | 0.354 | 18.50 (-5.81, 42.81) | 0.57 |
|  | PTG |  | 71.04 ± 41.82 | 89.91 ± 18.58 | 0.142 | 18.87 (-2.63, 40.37) | 0.58 |
|  | CG |  | 73.52 ± 32.69 | 74.76 ± 34.13 | > 0.999 | 1.24 (-17.91, 20.39) | 0.04 |
|  | All |  | 72.86 ± 36.95 | 84.33 ± 27.05 | 0.003* | 12.87 (4.63, 21.11) | 0.40 |
| Mental Health  (%) | AUG | 0.065 | 71.11 ± 16.15 | 78.22 ± 11.25 | 0.410 | 7.11 (-2.48, 16.70) | 0.50 |
|  | PTG |  | 71.65 ± 17.72 | 81.39 ± 10.21 | 0.013* | 9.74 (1.25, 18.23) | 0.69 |
|  | CG |  | 70.48 ± 14.60 | 71.59 ± 13.28 | > 0.999 | 1.10 (-6.45, 8.66) | 0.08 |
|  | All |  | 71.03 ± 15.85 | 76.51 ± 12.44 | < 0.001* | 5.99 (2.73, 9.24) | 0.42 |
| Health Evolution (%) | AUG | < 0.001* | 50.00 ± 14.85 | 77.78 ± 16.91 | < 0.001* | 27.78 (12.68, 42.88) | 1.60 |
|  | PTG |  | 53.26 ± 13.70 | 83.70 ± 20.79 | < 0.001* | 30.44 (17.08, 43.79) | 1.76 |
|  | CG |  | 45.69 ± 17.77 | 52. 59 ± 18.11 | > 0.999 | 6.90 (-5.00, 18.79) | 0.40 |
|  | All |  | 49.29 ± 15.91 | 69.29 ± 23.39 | < 0.001* | 21.70 (16.59, 26.82) | 1.25 |

AUG, Autonomous Group; GC, Control Group; CI, confidence interval; Inter, interaction (time/group); MC, mean change; PTG, Personal Trainer Group; SD, standard deviation.

*, significant difference.

## Supplementary Table S6. Between-group comparisons of mean change in dimensions of the SF-36 questionnaire.

| **Dimension** | **Group** | ***p*** | ***p ^A^*** | **MD (95% CI)** | **Cohen's *d*** | ***p ^B^*** | **MD (95% CI)** | **Cohen's *d*** | ***p ^C^*** | **MD (95% CI)** | **Cohen's *d*** |
| --- | --- | --- | --- | --- | --- | --- | --- | --- | --- | --- | --- |
| Physical  Function  (%) | AUG | 0.052 | 0.975 | -0.41  (-4.97, 4.16) | -0.07 | 0.149 | 3.47  (-0.93, 7.87) | 0.57 | 0.068 | 3.88  (-0.23, 7.98) | 0.64 |
|  | PTG |  |  |  |  |  |  |  |  |  |  |
|  | CG |  |  |  |  |  |  |  |  |  |  |
| Physical Role  (%) | AUG | 0.006* | 0.665 | -5.18  (-19.55, 9.19) | -0.27 | 0.100 | 11.95  (-1.75, 25.65) | 0.63 | 0.006* | 17.13  (4.37, 29.88) | 0.90 |
|  | PTG |  |  |  |  |  |  |  |  |  |  |
|  | CG |  |  |  |  |  |  |  |  |  |  |
| Bodily  Pain  (%) | AUG | 0.032* | 0.664 | -5.45  (-20.54, 9.65) | -0.28 | 0.282 | 9.06  (-5.11, 23.22) | 0.46 | 0.028* | 14.50  (1.31, 27.69) | 0.74 |
|  | PTG |  |  |  |  |  |  |  |  |  |  |
|  | CG |  |  |  |  |  |  |  |  |  |  |
| General Health  (%) | AUG | 0.008* | 0.221 | -6.03  (-14.64, 2.58) | -0.53 | 0.424 | 4.31  (-3.91, 12.53) | 0.38 | 0.005* | 10.34  (2.66, 18.03) | 0.91 |
|  | PTG |  |  |  |  |  |  |  |  |  |  |
|  | CG |  |  |  |  |  |  |  |  |  |  |
| Vitality  (%) | AUG | < 0.001* | 0.021* | -9.36  (-17.57, -1.16) | -0.86 | 0.002* | 11.60  (3.73, 19.47) | 1.07 | < 0.001* | 20.96  (13.62, 28.29) | 1.93 |
|  | PTG |  |  |  |  |  |  |  |  |  |  |
|  | CG |  |  |  |  |  |  |  |  |  |  |
| Social Function  (%) | AUG | 0.240 | 0.817 | -2.98  (-14.74, 8.78) | -0.19 | 0.617 | 4.40  (-6.82, 15.62) | 0.28 | 0.218 | 7.38  (-3.11, 17.87) | 0.47 |
|  | PTG |  |  |  |  |  |  |  |  |  |  |
|  | CG |  |  |  |  |  |  |  |  |  |  |
| Emotional Role  (%) | AUG | 0.015* | 0.973 | 1.61  (-15.85, 19.07) | 0.07 | 0.035* | 17.67  (1.02, 34.31) | 0.76 | 0.041* | 16.06  (0.56, 31.55) | 0.69 |
|  | PTG |  |  |  |  |  |  |  |  |  |  |
|  | CG |  |  |  |  |  |  |  |  |  |  |
| Mental Health  (%) | AUG | 0.003* | 0.605 | -2.94  (-10.29, 4.41) | -0.30 | 0.082 | 6.37  (-0.64, 13.37) | 0.65 | 0.003* | 9.31  (2.79, 15.83) | 0.96 |
|  | PTG |  |  |  |  |  |  |  |  |  |  |
|  | CG |  |  |  |  |  |  |  |  |  |  |
| Health Evolution (%) | AUG | < 0.001* | 0.674 | -4.89  (-18.67, 8.90) | -0.27 | < 0.001* | 23.83  (10.65, 37.01) | 1.31 | < 0.001* | 28.71  (16.26, 41.16) | 1.58 |
|  | PTG |  |  |  |  |  |  |  |  |  |  |
|  | CG |  |  |  |  |  |  |  |  |  |  |

AUG, Autonomous Group; CG, Control Group; CI, confidence interval; MD, mean difference; PTG, Personal Trainer Group.

*, significant difference. *p*, exercise modality; *p ^A^*, differences between the Autonomous Group and Personal Trainer Group; *p ^B^*, differences between the Autonomous Group and Control Group; *p ^C^*, differences between the Personal Trainer Group and Control Group.

## Supplementary Table S7. Effect of exercise on psychological vitality and affective state questionnaires (mean ± SD).

| **Variable** | **Group** | ***p* inter** | **PRE** | **POST** | ***p*** | **MC (95% CI)** | **Cohen's *d*** |
| --- | --- | --- | --- | --- | --- | --- | --- |
| Vitality | AUG | 0.043* | 4.02 ± 1.27 | 5.10 ± 1.03 | 0.001* | 1.08 (0.29, 1.87) | 0.89 |
|  | PTG |  | 4.27 ± 1.33 | 5.24 ± 1.07 | < 0.001* | 0.98 (0.28, 1.67) | 0.81 |
|  | CG |  | 3.94 ± 1.19 | 4.28 ± 1.29 | > 0.999 | 0.34 (-0.28, 0.96) | 0.28 |
|  | All |  | 4.07 ± 1.25 | 4.80 ± 1.23 | < 0.001* | 0.80 (0.53, 1.07) | 0.66 |
| Positive Affective State | AUG | 0.054 | 4.89 ± 1.46 | 5.68 ± 0.85 | 0.077 | 0.79 (-0.04, 1.62) | 0.62 |
|  | PTG |  | 4.95 ± 1.55 | 5.74 ± 0.96 | 0.025* | 0.79 (0.06, 1.53) | 0.62 |
|  | CG |  | 4.68 ± 1.29 | 4.76 ± 1.34 | > 0.999 | 0.08 (-0.58, 0.73) | 0.06 |
|  | All |  | 4.82 ± 1.41 | 5.33 ± 1.19 | < 0.001* | 0.56 (0.28, 0.84) | 0.44 |
| Negative Affective State | AUG | 0.001* | 2.78 ± 1.17 | 1.87 ± 1.00 | 0.022* | -0.91 (-1.75, -0.08) | -0.81 |
|  | PTG |  | 2.93 ± 1.38 | 1.81 ± 0.71 | < 0.001* | -1.11 (-1.85, -0.37) | -1.00 |
|  | CG |  | 2.77 ± 1.15 | 2.82 ± 1.16 | > 0.999 | 0.06 (-0.60, 0.71) | 0.05 |
|  | All |  | 2.82 ± 1.22 | 2.25 ± 1.09 | < 0.001* | -0.66 (-0.94, 0.37) | -0.59 |

AUG, Autonomous Group; CG, Control Group; CI, confidence interval; Inter, interaction (time/group); MC, mean change; PTG, Personal Trainer Group; SD, standard deviation.

*, significant differences.

## Supplementary Table S8. Between-group comparisons of mean change in psychological vitality and affective state questionnaires.

| **Variable** | **Group** | ***p*** | ***p ^A^*** | **MD (95% CI)** | **Cohen's *d*** | ***p ^B^*** | **MD (95% CI)** | **Cohen's *d*** | ***p ^C^*** | **MD (95% CI)** | **Cohen's *d*** |
| --- | --- | --- | --- | --- | --- | --- | --- | --- | --- | --- | --- |
| Vitality | AUG | 0.005* | > 0.999 | -0.01  (-0.74, 0.73) | -0.01 | 0.023* | 0.78  (0.08, 1.47) | 0.82 | 0.013* | 0.78  (0.13, 1.44) | 0.83 |
|  | PTG |  |  |  |  |  |  |  |  |  |  |
|  | CG |  |  |  |  |  |  |  |  |  |  |
| Positive Affective State | AUG | 0.002* | > 0.999 | -0.03  (-0.72, 0.66) | -0.04 | 0.009* | 0.82  (0.17, 1.48) | 0.92 | 0.003* | 0.83  (0.24, 1.47) | 0.96 |
|  | PTG |  |  |  |  |  |  |  |  |  |  |
|  | CG |  |  |  |  |  |  |  |  |  |  |
| Negative Affective State | AUG | < 0.001* | > 0.999 | 0.11  (-0.58, 0.79) | 0.12 | 0.002* | -0.96  (-1.61, -0.31) | -1.09 | < 0.001* | -1.07  (-1.67, -0.46) | -1.21 |
|  | PTG |  |  |  |  |  |  |  |  |  |  |
|  | CG |  |  |  |  |  |  |  |  |  |  |

AUG, Autonomous Group; CG, Control Group; CI, confidence interval; MD, mean difference; PTG, Personal Trainer Group.

*, significant differences. *p,* exercise modality; *p ^A^*, differences between the Autonomous Group and Personal Trainer Group; *p ^B^*, differences between the Autonomous Group and Control Group; *p ^C^*, differences between the Personal Trainer Group and Control Group.
